# Supplementary material for: Chagas disease screening in pregnant Latin American women: Adherence to a systematic screening protocol in a non-endemic country
Source: PLoS Negl Trop Dis. 2021 Mar 24;15(3):e0009281. doi: 10.1371/journal.pntd.0009281 (PMC8021187; doi:10.1371/journal.pntd.0009281)
Supplement: Attachment — Submitted filename: Def CH023 CCD YC CT compresse.pdf [file pntd.0009281.s003.pdf]

Maternal–fetal transmission of *Trypanosoma cruzi*

23

Y. Carlier[1,2] *and C. Truyens*[1]
[1]Université Libre de Bruxelles (ULB), Brussels, Belgium,
[2]Tulane University, New Orleans, LA, United States

Chapter Outline

From maternal–fetal transmission of *Trypanosoma cruzi* to congenital Chagas disease: definitions and limits 518
Epidemiological aspects of congenital infection with *Trypanosoma cruzi* 519
Situation in Latin American endemic countries 519
Globalization of congenital infection with *Trypanosoma cruzi* 519
Epidemiological features specific to congenital infection with *Trypanosoma cruzi* 519
Routes of maternal–fetal transmission of *Trypanosoma cruzi* 521
Possible transmission routes of *Trypanosoma cruzi* parasites 521
The hematogenous transplacental route: strengths and weaknesses of the trophoblastic barrier 522
Ex vivo/in vitro interactions between *Trypanosoma cruzi* and placenta 522
Histopathologic studies of placentas from infected or uninfected neonates 523
Interpretation attempt of *Trypanosoma cruzi*–placenta interactions 523
Timing of maternal–fetal transmission of *Trypanosoma cruzi* 524
Parasitic factors involved in transplacental transmission and development of *Trypanosoma cruzi* infection in fetuses/newborns 525
Maternal factors involved in transmission and development of *Trypanosoma cruzi* infection 526
Parasitic load during pregnancy and transmission of congenital infection 526
Immunity in pregnant women and transmission of congenital infection 527
Other maternal factors involved in transmission of congenital infection 528
Interactions between the maternal and fetal immune systems 529
Main biorelevant transferred molecules 529
Maternal immunological imprinting/priming of infant immune responses 530
Fetal/neonatal factors involved in the development of congenital Chagas disease 530
Parasite amounts in newborns and severity of congenital Chagas disease 530
Immune responses in newborns of *Trypanosoma cruzi*-infected mothers 532
Interpretation attempt of fetal/neonatal immune responses to *Trypanosoma cruzi* 533
Other fetal/neonatal factors susceptible to interfere with the development of congenital Chagas disease 534

American Trypanosomiasis Chagas Disease. DOI: http://dx.doi.org/10.1016/B978-0-12-801029-7.00024-1
Copyright © 2017 Elsevier Inc. All rights reserved.

Vertical transmission of *Trypanosoma cruzi* in other mammals 534
Vertical transmission in the natural mammal reservoir of *Trypanosoma cruzi* 534
Vertical transmission in experimental infection with *Trypanosoma cruzi* 534
Pregnancy outcomes, clinical manifestations, and long-term consequences of congenital Chagas disease 535
Trypanosoma cruzi infection and pregnancy outcomes 535
Clinical manifestations of congenital Chagas disease 536
Long-term consequences of congenital infection with *Trypanosoma cruzi* 537
Laboratory diagnosis of congenital infection with *Trypanosoma cruzi* 537
Detection of infection in pregnant women 538
In utero detection of fetal infection 538
Detection of neonatal infection (0–4 weeks after birth) 538
Infant detection of *Trypanosoma cruzi* congenital infection (1–12 months after birth) 540
Recommendations 541
Treatment of congenital infection with *Trypanosoma cruzi* 541
Prevention and control of congenital *Trypanosoma cruzi* infection 542
Conclusions 544
References 544

From maternal–fetal transmission of *Trypanosoma cruzi* to congenital Chagas disease: definitions and limits

Maternal–fetal *transmission* of *Trypanosoma cruzi* induces congenital infection (from its Latin etymology "cum" (with), and "genitus" (engendered)) when transmitted live parasites persist and multiply in fetuses and/or after birth (*development of infection*). Maternal–fetal transmission can be "prenatal" (in utero) or "perinatal" (at the time of delivery). It excludes the "postnatal" transmission of parasites (mainly through maternal milk by breast feeding) and the transmission of dead parasites, parasitic DNA, or other molecules released from parasites in the mother and likely to be found in fetal blood.

The terms "mother-to-child transmission" or "vertical transmission" have a broader connotation corresponding to transmission from one generation to the next, including prenatal, perinatal, as well as postnatal transmission of live parasites. The term "congenital infection with *T. cruzi*" refers to asymptomatic as well as symptomatic cases of infection, whereas the term "congenital Chagas disease" should be used only for symptomatic cases, though, often, both terms are confounded. In order to facilitate the reading of this chapter, the terms "mother" or "maternal" will be also used for pregnant women (before delivery), albeit such terms normally refer to the status of women having delivered.

The present chapter updates the previously one published in the first edition of the book in 2010.[1] The critical review of interactions between *T. cruzi* parasites, pregnant women, placenta, and fetuses recently published by the authors[2] can usefully complete the reading of the present chapter.

Epidemiological aspects of congenital infection with *Trypanosoma cruzi*

Situation in Latin American endemic countries

Historically, Carlos Chagas himself, in 1911, referred to the possibility of congenital transmission of *T. cruzi* infection when he found trypomastigotes in the blood smear of a 2-month-old child whose mother was infected.[3] Dao, in 1949, was the first in reporting cases of congenital Chagas disease from Venezuela.[4]

According a recent WHO estimation,[5] 1,125,000 women in fertile age are infected with *T. cruzi* in the 21 Latin American countries where Chagas disease is currently endemic. The incidence of congenital infection is estimated to be 8668 cases per year, with nearly 50% of such cases grouped in Mexico, Argentina, and Colombia. Congenital transmission would be responsible for 90 to 100% of new annual cases of infection in countries declared free of transmission by the vector *Triatoma infestans* (such as Brazil, Chile, Uruguay) and for 6 to 90% in other countries depending on the achievement of their vector control programs (e.g., Colombia: 16.5%; Mexico: 22.6%; Argentina: 57.5%) (Table 23.1).

The maternal–fetal transmission rate, classically defined as the number of congenital cases/number of *T. cruzi*-infected mothers, can gain in precision by including multiple births if expressed as the number of congenitally infected infants divided by the number of infants born to infected mothers. Well defined, it occurs in an average of 4.7% of chronically infected mothers in endemic areas,[6] though differences can be observed between countries.[7,8]

Globalization of congenital infection with Trypanosoma cruzi

Migration of Latin American people particularly during these last decades has promoted Chagas disease as a global disease, now observed in areas deprived from vector transmission (Europe, Canada, Japan, Australia) or with exceptional vector transmission to humans, such as the United States. The current trend toward the feminization of such migration[9] is relevant for an increased risk of congenital transmission into nonendemic areas. Cases of congenital Chagas disease have been reported mainly in Spain,[10,11] but also in Sweden,[12] Switzerland,[13] and more recently in the United States,[14] Canada,[15] and Japan.[16] The congenital route is the main transmission mode of *T. cruzi* in nonendemic areas, over blood transfusions and organ transplantations (occurring in an average of 2.7% of chronically infected mothers[6]).

Epidemiological features specific to congenital infection with Trypanosoma cruzi

Congenital transmission of *T. cruzi*, in contrast with toxoplasmosis, can arise in both acute and chronic phases of maternal infection and be repeated at each pregnancy, i.e., throughout the fertile period of woman life (most cases of congenital infection derive from chronically infected mothers, having been infected by insect

Table 23.1 **WHO estimations of women in fertile age infected with *T. cruzi* and congenital cases in Latin American countries for the year 2010[5]**

Country	Population	Prevalence of infection (%)	Nb of infected women aged 15–44 years	Congenital cases		
				Annual nb	Incidence (%)	Annual new infection cases[b] (%)
**South America**						
Argentina	41,343,000	3.640	211,102	1 457	0.210	57.5
Bolivia	9,947,000	6.104	199,351	616	0.235	7.1
Brazil	190,755,799	0.030	119,298	571	0.020	92.5
Chile	17,095,000	0.699	11,771	115	0.046	100.0
Colombia	48,805,000	0.956	116,221	1046	0.114	16.5
Ecuador	14,483,499	1.379	62,898	696	0.317	25.4
Guyanas[a]	1,501,962	0.838	3818	18	0.075	6.0
Paraguay	8,668,000	2.130	63,385	525	0.340	63.9
Peru	28,948,000	0.439	28,132	232	0.038	10.1
Uruguay	3,301,000	0.237	1858	20	0.040	100.0
Venezuela	27,223,000	0.710	40,223	665	0.110	43.2
**Central America**						
Belize	315,000	0.330	272	25	0.333	71.4
Costa Rica	4,516,000	0.169	1728	61	0.080	85.9
El Salvador	6,952,000	1.297	18,211	234	0.187	19.4
Guatemala	13,550,000	1.230	32,759	164	0.035	11.4
Honduras	7,989,000	0.917	16,149	257	0.126	21.8
Nicaragua	5,604,000	0.522	5822	138	0.124	26.5
Panama	3,557,687	0.515	6332	40	0.056	18.6
**North America**						
Mexico	112,468,855	0.779	185,600	1788	0.089	22.6
Total	543,877,115	1.055	1,124,930	8 668	0.089	22.5

Nb/nb=number
[a]including French Guyana, Guyana and Surinam.
[b]calculated from WHO data[5] as: estimated annual number of congenital infection cases/estimated annual infection cases due to congenital + vector transmissions.

vectors since childhood by residing in endemic areas of Latin America).[1,17] Moreover, transgenerational (vertical) transmission of parasites can occur from an infected mother to her daughter who in turn transmits parasites to her own infants, etc. All these elements can contribute to the familial clustering of congenital cases.[18,19] Such epidemiological features specific to *T. cruzi* infection suggest a particularly long-term risk of mother-to-offspring transmission through the pool of currently infected pregnant women in endemic, as well as in nonendemic areas.

This pinpoints congenital infection with *T. cruzi* as an important public health problem which can easily extend in space (through migrations of infected fertile women) and time (for the reasons mentioned above).[1,20]

Routes of maternal–fetal transmission of *Trypanosoma cruzi*

Possible transmission routes of Trypanosoma cruzi *parasites*

A possible mode of maternal–fetal transmission might be through parasites released into amniotic fluid (AF) (Fig. 23.1; see section: Histopathologic studies of placentas from infected or uninfected neonates).[21,22] The latter might contaminate the fetuses by oral or pulmonary routes, or eventually by skin penetration (fetuses are bathing in AF and continuously absorbing it). Lung and skin infections with *T. cruzi* have been reported in macerated fetuses and stillborns.[23,24] However, AF contains potent antimicrobial peptides[25] and *T. cruzi* parasites are not found by microscopic observation and parasitic DNA is hardly detected in AF or aspirated gastric fluid content of infected asymptomatic newborns.[26,27]

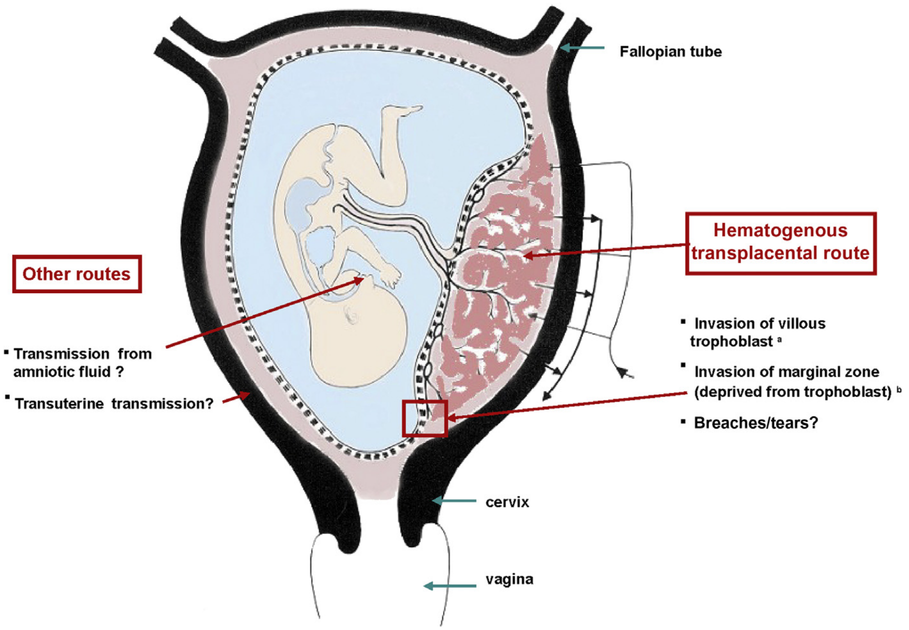

Figure 23.1 Possible routes of maternal–fetal transmission of *T. cruzi*. [a]In case of huge parasite amounts in the placental intervillous space; [b]in case of moderate parasite amounts in the placental intervillous space.
Source: According to Carlier Y, Truyens C. Maternal-fetal transmission of Trypanosoma cruzi. In: Telleria J, Tibayrenc M, editors. *American trypanosomiasis-Chagas disease. One hundred years of research*. London, Burlington: Elsevier; 2010. p. 539–81.

The possibility of placental/fetal invasion directly from the uterine wall (transuterine route) remains to be determined, since *T. cruzi* amastigote nests are observed in placental deciduas of mothers having delivered infected neonates[28] (Fig. 23.1).

Although outside of the scope of this chapter, it is interesting to mention the possible postnatal transmission of parasites through maternal milk by breast feeding. Only one report mentions a possible infant contamination during lactation.[29] Another one indicates the presence of parasites in milk during maternal infection,[30] but their detection might have been related to a contamination of collected milk with maternal blood.[31] Others studies do not observe parasites in milk of infected women.[32–34] Moreover, blood trypomastigotes, by contrast with metacyclic trypomastigotes expressing Tcgp82, can hardly adhere and migrate through the gastric mucin layer and survive into the gastric milieu.[35,36]

Whether fetal contamination with parasites coming from AF or uterine wall, and/or eventually by breast feeding remains a possibility in acute or reactivated infection,[37] these routes are unlikely in chronic infection.

Placenta is therefore the key fetal organ facing the parasites present into the intervillous blood space and from which transmission depends. Such a route requires parasites to cross or skirt the trophoblastic barrier (first placental line of defense) (Fig. 23.1).

The hematogenous transplacental route: strengths and weaknesses of the trophoblastic barrier

Villi of the human placenta are covered by two layers of trophoblast: an outer layer called syncytiotrophoblast and an inner layer termed cytotrophoblast ("Langhans" cells). Extravillous cytotrophoblast cells also cover nonvillous structures (e.g., the chorionic plate) and migrate from the placenta into the uterine wall. To a great degree, only the syncytiotrophoblast is interposed between maternal blood and fetal tissues, since the cytotrophoblast cell number decreases during the gestation period.[38]

Besides the physical barrier formed by the syncytiotrophoblast that forgoes intercellular junctions, the placenta is an active immunological organ also able to initiate an innate immune response. It can release pro-inflammatory cytokines, chemokines, reactive oxygen- and nitrogen-intermediates (NO), and antimicrobial peptides,[39] through Toll-like receptors (TLRs) recognizing pathogen-associated molecular patterns (PAMPs).[40]

However, the placental barrier displays some weaknesses since some areas are not covered by trophoblast, such as the marginal zone joining the membranes to the chorionic and basal plates. The latter is constituted with smooth muscle cells embedded in an extracellular fibrinoid matrix (fibronectin, collagen, laminin), only covered by a nontrophoblastic epithelium.[41]

Ex vivo/in vitro interactions between Trypanosoma cruzi *and placenta*

Ex vivo studies show the inhibiting role of placental subfractions on trypomastigote infectivity,[42] the slow multiplication of parasites in villous explants and their killing

by NO produced by placental cells,[43] arguing for an activation of placental innate defenses in the presence of parasites. Other studies show that low concentrations of parasites induce proliferation, differentiation, and finally a turnover of the syncytiotrophoblast of such explants, considered as another mechanism to clear parasites out of the placental environment.[44] However, high concentrations of parasites induce massive destruction of the trophoblastic cells of villi by infecting them. *T. cruzi* attaches to trophoblastic cells through interactions with calreticulin and C1q and induces a disassembly of actin in cytosqueletton, the placental alkaline phosphatase contributing to such invasion.[45–47]

Histopathologic studies of placentas from infected or uninfected neonates

In placentas of severe and mortal cases of congenital Chagas disease (abortions, stillbirths, or premature newborns dead in the neonatal period; rarely observed today), a severe placentitis/villitis occurs with large areas of trophoblast destruction and necrosis. Amastigote parasites are found in villous trophoblast and stromal cells.[48,49] Immunohistochemical studies show important inflammatory responses in such placentas, with infiltrates mainly composed of $CD68^+$ macrophages, $CD8^+$ T lymphocytes, and few natural killer (NK) cells, and an intense production of TNF-α.[50]

In placentas of live neonates suffering mild congenital *T. cruzi* infection (frequent today), villitis is much less marked or not observed (despite some degree of reorganization of extracellular matrix). Parasites are not, or hardly, identified in villous and extravillous trophoblast. Necrosis and lysis associated with infiltration of neutrophils and lymphocytes are more frequently detected in chorionic plate and membranes surrounding the fetus (chorioamnionitis) and umbilical cord (funisitis).[28,50,51] In serial biopsies performed in 19 placentas from infected live Bolivian newborns, we observed an unexpected high density of parasites at the level of marginal zone, joining the membranes to the chorionic and basal plates, with gradually decreasing densities in the chorionic plate and distant membranes.[1,22]

Histopathology of placentas of uninfected neonates born to infected mothers is similar to that of control newborns of uninfected mothers (or display slight inflammation without lymphocyte infiltration).[17,28]

Interpretation attempt of Trypanosoma cruzi*–placenta interactions*

Based on the observations mentioned above, we can depict four scenarios of interactions between the parasite and the placenta, considering the importance of placental lesions and the occurrence of transmission (Fig. 23.1).

1. The huge parasite load in the intervillous space overflows the placental defenses, leading to infection and rupture of the trophoblastic barrier. This results in the transmission of large quantities of free trypomastigotes as well as of amastigote-infected cells, and to severe and mortal congenital Chagas disease. In such a situation, the placental inflammation (placentitis) is strongly marked and has pathological instead of protective effects,

with the substantial release of TNF-α contributing to the trophoblast rupture, as well as to fetal and neonatal mortality.[52] The high local production of other inflammatory mediators, such as reactive oxygen species, NO and peroxynitrite, has deleterious effects on placental vascularization.[53] This is probably what occurs in acute or reactivated infection during pregnancy displaying the highest parasitemias (see section: Parasitic load during pregnancy and transmission of congenital infection).

2. In the presence of moderate parasite amounts in the intervillous space, the placental innate defenses are only slightly activated. Indeed, *T. cruzi*, being partially deficient in strong PAMPs,[54] can hardly stimulate the TLRs expressed on the trophoblast.[40] This does not induce the rupture of the trophoblastic barrier and transmission can occur by the marginal zone deprived of trophoblast (see section: Histopathologic studies of placentas from infected or uninfected neonates[41]). Parasites invade cells of this zone, and those surviving the mesenchymal defense (second placental line of defense) can easily spread by successive infections of fibroblasts, myofibroblasts, and macrophages (Hofbauer cells) within the chorion. They finally infect myocytes and endothelial cells lining fetal vessels embedded in chorionic plate or umbilical cord, and gain access to the fetal circulation.[1,22] This situation likely corresponds to the common mild congenital *T. cruzi* infections observed in roughly 5% of live newborns of chronically infected women (see section: Situation in Latin American endemic countries). Additionally, the infection spreads in membranes surrounding the fetus, inducing their embrittlement. This leads to their premature rupture and contribute to the premature birth, frequently observed in these cases.[55]
3. Parasite transmission (of trypomastigotes as well as amastigote-infected cells) occurs at delivery, when placental breaches/tears appear naturally with labor contractions. This route, which can be avoided by cesarean delivery (see section: Timing of maternal–fetal transmission of *Trypanosoma cruzi*) is used for transmission of HIV-infected leukocytes.[56] It is independent of the activation of placental innate defenses.
4. There is neither parasite transmission, nor disruption of the trophoblastic barrier, nor inflammation; placental innate defenses are not activated by the too low blood parasite amount; this might concern the 95% of chronically infected pregnant women displaying weak parasitemias (see section: Parasitic load during pregnancy and transmission of congenital infection) and delivering uninfected newborns.

A placental microbiome has been recently discovered.[57] Its role in the transmission of congenital infection deserves further investigations.

Mutations in the placental genes for the disintegrin and metalloproteinases ADAM12 and MMP2 are associated to the susceptibility to congenital infection with *T. cruzi*.[58] Whether such mutations in placental genes are sufficiently frequent to contribute to transmission remains to be determined.

Timing of maternal–fetal transmission of *Trypanosoma cruzi*

There is likely little or no transmission of blood trypomastigotes during the first trimester of pregnancy, since the placental intervillous space is not open. Maternal blood supply becomes continuous and diffuse in the entire placenta only after the 12th week of gestation.[59] The absence of developmental malformations in live

newborns congenitally infected with *T. cruzi* (see section: Clinical manifestations of congenital Chagas disease) also suggests there is no transmission at the early stages of organogenesis in the embryo.

Abortions, stillbirths, and premature births in *T. cruzi*-infected women are more frequent for gestational ages between 19 and 37 weeks of pregnancy, but the proof of congenital infection as responsible for such pejorative outcomes has not been systematically investigated.[51,60] The rare reported cases of acute *T. cruzi* infection during pregnancy indicate possible transmission around the 20th week of pregnancy.[61] However, in most pregnant women who are in the chronic phase of infection (see section: Epidemiological features specific to congenital infection with *Trypanosoma cruzi*), it is impossible to pinpoint the timing of maternal−fetal transmission of *T. cruzi*. Transmission rates in vaginal and cesarean deliveries of infected women are similar (see Ref. [62], unpublished own data), indicating that most transmissions are prenatal, though possible later additional perinatal transmission during labor cannot be excluded (see section: Interpretation attempt of *Trypanosoma cruzi*−placenta interactions).

Parasitic factors involved in transplacental transmission and development of *Trypanosoma cruzi* infection in fetuses/newborns

Based on different molecular approaches, *T. cruzi* has been classified into six phylogenetic lineages, so-called "discrete typing units" (DTUs) named TcI to TcVI,[63] though recent studies propose to reconsider such classification into only three reliable mitochondrial clades.[64]

Five *T. cruzi* DTUs (TcI, TcII, TcIII, TcV, and TcVI) and associations of them (coinfections) have been identified in human cases of congenital infection. The DTU TcV has been reported in 80−100% of congenital cases in Argentina, Bolivia, Southern Brazil, Chile, and Paraguay.[65−74] DTUs TcII and TcVI have been reported in neonates from Argentina, Bolivia, Brazil (Bahia state), and Chile,[65,67,71,73,75] whereas TcIII has been identified in Paraguay[76] and TcI in some neonates from Argentina, Chile, and Colombia.[71,72,77]

The distribution of DTUs identified in congenital cases is similar to that found in the local population.[65,67,68] The same DTUs are generally detected in mothers and their infected newborns, as well as in congenitally infected twins or siblings born in consecutive gestations.[66,67] However, when multiclonal infections occur with different DTUs or DTU variants,[65,67,68,70,71,73,74] slight differences can be observed between clones infecting mothers and those found in congenitally infected infants, suggesting that a natural selection of transmitted parasite populations might occur,[67,71,74] perhaps through interactions with placental cells and/or the host immune system (see sections: Interpretation attempt of *Trypanosoma cruzi*−placenta interactions and Immunity in pregnant women and transmission of congenital infection). So, at this time, there is no clear evidence of a relationship between *T. cruzi* DTUs (defined by the currently used molecular markers) and congenital infection in humans.

Maternal factors involved in transmission and development of *Trypanosoma cruzi* infection

Parasitic load during pregnancy and transmission of congenital infection

A lot of data has now well established that blood parasite amount during pregnancy is a key factor for congenital transmission. Although parasitemia slightly increases during pregnancy (on second and third trimesters),[78,79] congenital transmission during chronic infection increases with parasitemias and occurs mainly in women displaying around 10–20 p/mL (i.e., 10–20 times higher than in non-transmitting women.[62,66,80–84] Transmission rates are much higher in acute infection (in 6/11–54% of reported cases in acutely infected mothers[17,61]) and reactivated Chagas disease (100%, in case of coinfection with HIV[85,86]), displaying extremely high and huge parasitemias, respectively (Fig. 23.2).

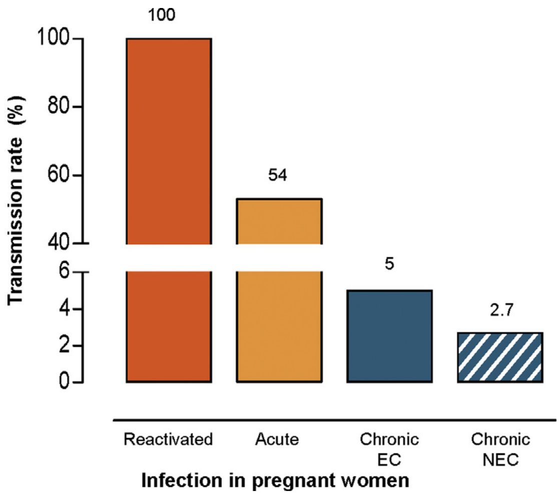

Figure 23.2 Maternal–fetal transmission rates of *T. cruzi* infection, according to the clinical phase/form of infection in pregnant women.
Source: Reactivated Chagas disease: data according to Freilij H, Altcheh J, Muchinik G. Perinatal human immunodeficiency virus infection and congenital Chagas' disease. Pediatr *Infect Dis* 1995;**14**:161–162 and Scapellato PG, Bottaro EG, Rodriguez-Brieschke MT. Mother-child transmission of Chagas disease: could coinfection with human immunodeficiency virus increase the risk? *Rev Soc Bras Med Trop* 2009;**42**:107–109; acute Chagas disease: data according to Bittencourt AL. Possible risk factors for vertical transmission of Chagas' disease. *Rev Inst Med Trop Sao Paulo* 1992;**34**:403–408 and Moretti E, Basso B, Castro I, Carrizo P, Chaul M, Barbieri G, et al. Chagas' disease: study of congenital transmission in cases of acute maternal infection. *Rev Soc Bras Med Trop* 2005;**38**:53–55; chronic Chagas disease: average transmission rates in endemic and nonendemic countries (EC and NEC, respectively) according to Howard EJ, Xiong X, Carlier Y, Sosa-Estani S, Buekens P. Frequency of the congenital transmission of Trypanosoma cruzi: a systematic review and meta-analysis. *Brit J Obst Gyn* 2014;**121**:22–33.

Immunity in pregnant women and transmission of congenital infection

Maternal immunity is likely a factor limiting transmission of infections in fetus/neonate. *T. cruzi*-specific IgG antibodies play a protective role by favoring the clearance of circulating parasites through activated phagocytes expressing FcR, likewise contributing to limiting parasitemia (reviewed in Chapter 25, Protective host response to *Trypanosoma cruzi* and its limitations).

Analyses of innate immune defences in pregnant women by us and others show that monocytes from transmitting women display a less activated phenotype and release less TNF-α as compared to non-transmitting subjects.[80,87,88] Conversely, we also showed that blood cells from non-transmitting women produce higher levels of TNF-α, IL-1β, and IL-6 in response to *T. cruzi* or LPS/PHA than uninfected controls.[89]

As expected, infected pregnant women display an adaptive response to parasites with higher blood cell production and circulating levels of IFN-γ than in non-infected ones.[80,89,90] However, T cells from those transmitting *T. cruzi* to their fetuses produce three times less IFN-γ than cells from non-transmitting women (Fig. 23.3), which probably contributes to increase their parasitemia[80] (see section: Parasitic load during pregnancy and transmission of congenital infection).

Altogether, and as schematically shown in Fig. 23.4, such results indicate that (1) a strong innate immune response is associated with the absence of congenital transmission (in non-transmitting women); (2) women transmitting congenital infection present

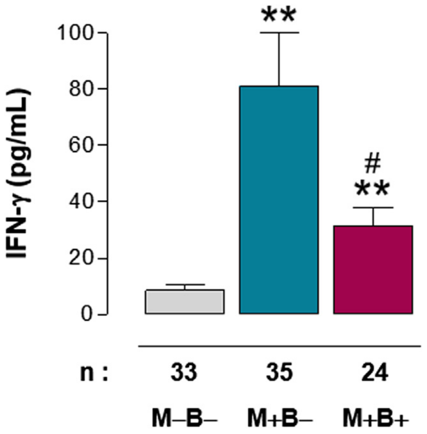

Figure 23.3 IFN-γ production by whole blood cells of mothers either noninfected (M−) or infected (M+) having delivered infected neonates (B+) or uninfected newborns (B−). Cells were stimulated with *T. cruzi* lysate for 24 h; IFN-γ levels were determined in supernatants of whole blood cells by ELISA; results are expressed as arithmetic means ± SEM; $^{**}p < 0.01$ versus M−B−; $\#p < 0.05$ M+B+ versus M + B − . *Source*: Data from Hermann E, Truyens C, Alonso-Vega C, Rodriguez P, Berthe A, Torrico F, et al. Congenital transmission of *Trypanosoma cruzi* is associated with maternal enhanced parasitemia and decreased production of interferon-gamma in response to parasite antigens. *J Infect Dis* 2004;**189**:1274−81.

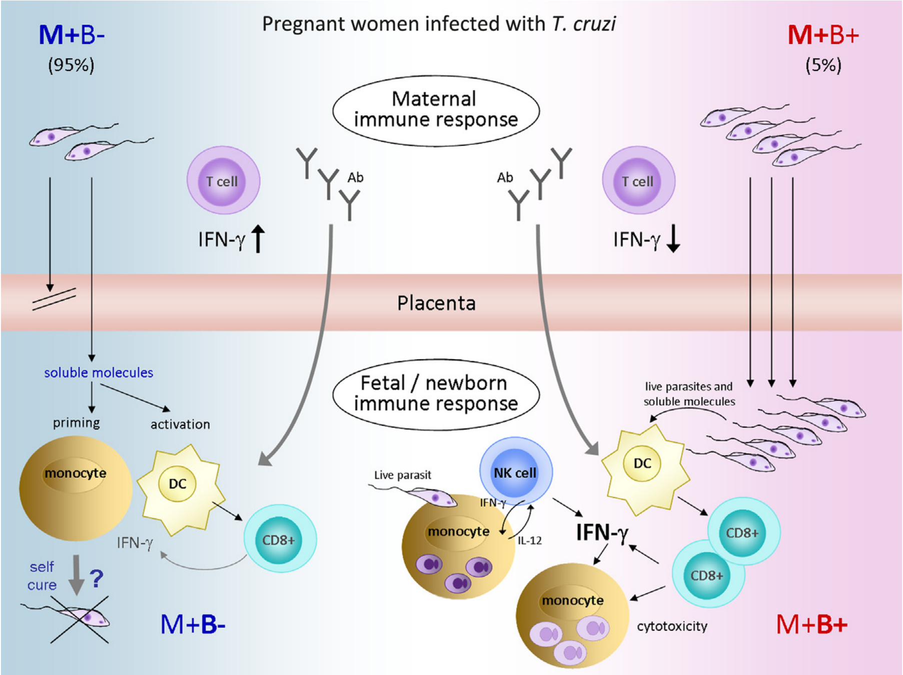

Figure 23.4 Schematical view of immune responses in infected pregnant women transmitting (M+B+) or not (M+B−) parasites and their fetuses according to the occurrence (M+B+) or not of congenital infection (M+B−).

some defect of their innate and adaptive immune responses, and (3) such defect mainly relies on a lower capacity to produce IFN-γ in response to *T. cruzi*.

Other maternal factors involved in transmission of congenital infection

The fact that around 95% of chronically infected pregnant women do not transmit parasites to their fetuses (see section: Situation in Latin American endemic countries) suggests that the physiological immune environment of pregnancy is likely not contributing to the immune defect of transmitting women. The pro-inflammatory environment occurring at different stages of gestation[91] might rather reboost the innate responses during chronic infection to keep infected women away from congenital transmission. However, as observed by our team,[55] but not by others,[62] primiparity (and young age) might be an additional factor contributing to parasite transmission.

Malnutrition and poverty also favor the congenital transmission of *T. cruzi*,[17,55,92,93] probably by worsening the deficiency of immune responses during pregnancy.[94] These factors might play a role in the difference observed between transmission rates in endemic and nonendemic countries (5% vs 2.7%, respectively[6]).

Maternal coinfection with *T. cruzi* and HIV results in increasing frequency and severity of congenital Chagas disease (see section: Pregnancy outcomes, clinical manifestations, and long-term consequences of congenital Chagas disease),[85,86] highlighting

the important role of maternal immunity and high parasitemia in favoring parasite transmission to the fetus (see sections: Parasitic load during pregnancy and transmission of congenital infection; Immunity in pregnant women and transmission of congenital infection). Interestingly, in vitro infection of human placenta with *T. cruzi* reduces HIV replication.[95]

The role of maternal genetic factors in favoring parasite transmission remains to be explored. Indeed, the familial clustering of congenital cases (see section: Epidemiological features specific to congenital infection with *Trypanosoma cruzi*),[18,19] as well as the persistence after delivery of the defective capacity of IFN-γ response to parasites in transmitting mothers (see section: Immunity in pregnant women and transmission of congenital infection),[80] suggest that some mothers might display a "transmitter" phenotype.

The diversity of intestinal microbiota and its impact on maternal immune responses has not been studied yet, nor has the role of the mode of delivery in transmitting it to the neonate. A cesarean section instead of a vaginal delivery prevents the transmission of the maternal vaginal and gut microbiota (recognized as essential immune-modulating factors in neonates and infants).[96] Particularly, the gut microbiota induces the production of anti-Gal antibodies which are lytic for *T. cruzi*[97] (reviewed in Chapter 25, Protective host response to *Trypanosoma cruzi* and its limitations).

Interactions between the maternal and fetal immune systems

Main biorelevant transferred molecules

Besides transmission of parasites resulting in congenital infection, other biologically relevant elements can be transferred to congenitally infected as well as uninfected fetuses from infected pregnant women.

The transplacental transfer of maternal IgG through the FcRn expressed on trophoblast[98] occurs in Chagas disease as in other infections.[99–102] The levels of *T. cruzi*-specific-antibodies in neonates and their repertoire are similar to those of their mothers,[100] indicating that the mild and focused placentitis often observed in chronically infected pregnant women (see sections: Histopathologic studies of placentas from infected or uninfected neonates; Interpretation attempt of *Trypanosoma cruzi*–placenta interactions) does not affect their transfer. Such transferred antibodies can persist up to 8–9 months after birth.[103–105]

Parasitic-circulating antigens (excreted and secreted), parasitic-breakdown molecules with pro-inflammatory activity and/or antigen surrogates (such as antibody idiotypes) are likely transferred through the placenta, as suggested in human and experimental infections with *T. cruzi*.[101,106–108]

This is probably also the case for parasitic-breakdown DNA (from dead or live parasites)[109,110] and the recently discovered small RNAs which are secreted by parasites as extracellular vesicles.[111] Indeed, transmission of maternal cells (microchimerism) and circulating cell-free DNA (ccfDNA) to fetuses is known to occur in normal pregnancy and to persist into adulthood.[112–114]

Except for IL-6, contradictory results have been reported on maternal−fetal transfer of cytokines.[115]

Maternal immunological imprinting/priming of infant immune responses

Besides the direct effects of transferred antibodies, the elements mentioned above (see section: Main biorelevant transferred molecules) can imprint/prime the fetal/neonatal immune system with long-term unexpected consequences (epigenetic effects). They can promote specific immunological memory or, conversely, immune tolerance, increasing resistance or susceptibility to further *T. cruzi* (re)infection, respectively (see section: Fetal/neonatal factors involved in the development of congenital Chagas disease), leading to more or less severe clinical forms of Chagas disease. Moreover, this imprinting in early life can also have consequences for responses toward other infections or vaccinations (reviewed in Refs. [116,117]). Our team showed that uninfected infants from infected mothers displayed stronger type 1 response to BCG (producing IFN-γ) and that congenitally infected infants developed this stronger response to hepatitis B, diphtheria, and tetanus vaccines, as well as enhanced antibody production to hepatitis B vaccine, compared to controls from uninfected mothers.[118] This might be related to an activation of fetal/neonatal dendritic cells (DCs) (induced by soluble parasite-derived molecules transferred from their mother).[119,120]

Fetal/neonatal factors involved in the development of congenital Chagas disease

Parasite amounts in newborns and severity of congenital Chagas disease

Parasitemias in congenitally infected newborns depend on: (1) the amount of parasites transmitted from the mothers (see section: Parasitic load during pregnancy and transmission of congenital infection); (2) the capacity of the infecting parasite strain(s) to invade and multiply into cells/tissues (virulence) and/or limit the efficiency of immune responses in fetuses/neonates; (3) the time allowed for parasite multiplication from the transmission time point during pregnancy (see section: Timing of maternal−fetal transmission of *Trypanosoma cruzi*);[72] and, finally (4) the amplitude of the own individual fetal/neonatal innate and/or adaptive immune responses against parasites, modulated according to the maternal imprinting/priming (see section: Maternal immunological imprinting/priming of infant immune responses). Parasitemias in newborns are generally higher than in their mothers and can reach more than 60,000 p/mL at birth (umbilical cord), though, in some cases, parasites are more easily detectable weeks or months after birth[66,72,82,103,121,122] (Fig. 23.5). Indeed, neonatal parasitic loads increase up to 1−3 months after birth, before decreasing as infection enters in the chronic phase.[103] As shown in Fig. 23.6, morbidity and mortality of congenital disease are associated with the highest parasitemias.[122]

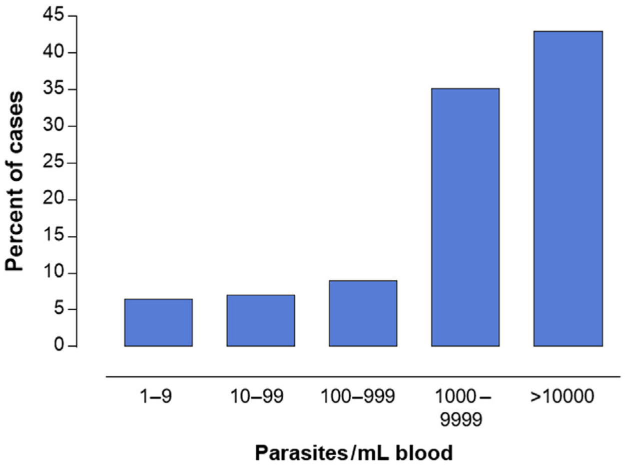

Figure 23.5 Distribution of parasitemias in Bolivian newborns congenitally infected with *T. cruzi*. Parasitemias were determined by qPCR.
Source: Data from Virreira M, Truyens C, Alonso-Vega C, Brutus L, Jijena J, Torrico F, et al. Comparison of *Trypanosoma cruzi* lineages and levels of parasitic DNA in infected mothers and their newborns. *Am J Trop Med Hyg* 2007;**77**:102–6.

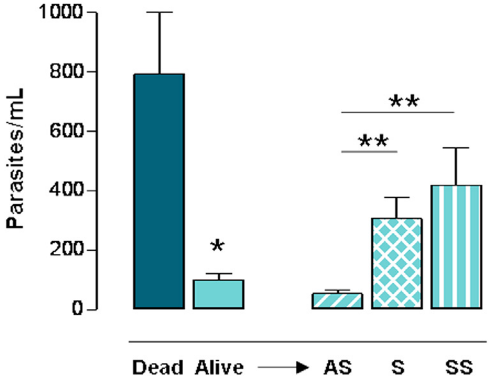

Figure 23.6 Mean parasitemias, morbidity and mortality at birth in Bolivian newborns congenitally infected with *T. cruzi*. *AS*, asymptomatic; *S*, symptomatic; *SS*, severe symptoms. Parasitemias were determined by microscopic examination of blood collected in microhematocrit tubes; results are expressed as geometric means; $^{*}p < 0.05$; $^{**}p < 0.01$.
Source: Data from Torrico F, Alonso-Vega C, Suarez E, Rodriguez P, Torrico MC, Dramaix M, et al. Maternal *Trypanosoma cruzi* infection, pregnancy outcome, morbidity, and mortality of congenitally infected and non-infected newborns in Bolivia. *Am J Trop Med Hyg* 2004;**70**:201–9; Torrico MC, Solano M, Guzman JM, Parrado R, Suarez E, Alonzo-Vega C, et al. Estimation of the parasitemia in *Trypanosoma cruzi* human infection: high parasitemias are associated with severe and fatal congenital Chagas disease. *Rev Soc Bras Med Trop* 2005;**38**(Suppl 2):58–61.

Immune responses in newborns of Trypanosoma cruzi-*infected mothers*

T. cruzi-congenitally infected newborns display lower circulating levels of TNF-α, IFN-γ, and IL-18 compared to uninfected neonates of infected mothers,[88,123,124] as well as a decreased proportion of CD56^{bright} NK cells (innate immune response).[125] By contrast, we have also shown that such neonates can mount a strong *T. cruzi*-specific T cell response (adaptive immune response) with activated CD8 T cells having cytotoxic activities and producing IFN-γ, but not IL-4.[126] They stimulate also a B cell response producing IgG, IgM, and IgA antibodies.[127–129] However, as shown in Fig. 23.7, the capacity of blood cells (mainly T cells) of congenitally infected neonates to produce IFN-γ in response to *T. cruzi*, is variable from one neonate to another, and inversely related to their parasitemias and the severity of infection (see section: Parasite amounts in newborns and severity of congenital Chagas disease),[2] confirming the key role of IFN-γ in controlling the severity of *T. cruzi* infection (see section: Immunity in pregnant women and transmission of congenital infection), as previously reported in adult subjects.[130]

By contrast, uninfected neonates from infected mothers display an unexpected strong inflammatory status. We and others showed that their blood cells are activated and produce higher levels of IL-1β, IL-6, and TNF-α, in response to *T. cruzi* or LPS/PHA than control uninfected neonates born to uninfected

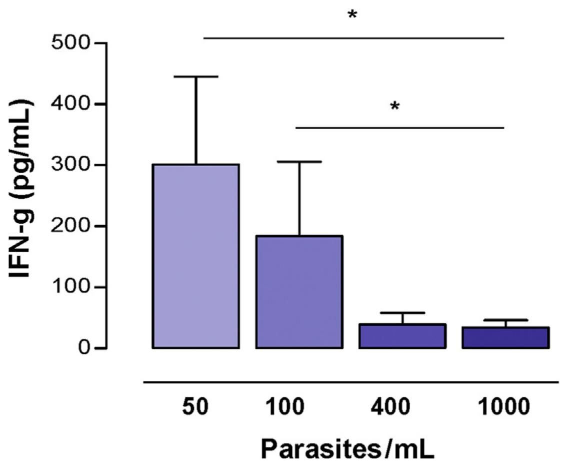

Figure 23.7 IFN-γ production by whole blood cells of congenitally infected neonates according to their parasitemias at birth. Cells were stimulated with *T. cruzi* lysate for 24 h; IFN-γ levels were determined in supernatants of whole blood cells by ELISA; results are expressed as arithmetic means ± SEM; $*p < 0.05$; parasitemias were determined by microscopic examination of the buffy coat from blood collected in microhematocrit heparinized tubes. *Source*: According to Carlier Y, Truyens C. Congenital Chagas disease as an ecological model of interactions between *Trypanosoma cruzi* parasites, pregnant women, placenta and fetuses. *Acta Trop* 2015;**151**:103–15.

mothers,[88–90,101] whereas they display no or low degrees of specific T cell activation and responses to *T. cruzi* antigens or surrogates.[101,106,131] However, their B cell responses seem more stimulated with the production of IgA and overall IgM antibodies.[1,101,129] Since live parasites have, apparently, not entered in contact with these fetuses/neonates, such cell activation and responses result from transmission of maternally derived molecules (see section: Interactions between the maternal and fetal immune systems).

Altogether, and as schematically shown in Fig. 23.4, such results indicate that (1) more inflammation (innate immune response) than specific T cell response is observed in uninfected newborns from infected mothers, such status deriving from maternal priming; (2) congenitally infected neonates display weak innate immune responses, but stronger T cell responses, mainly based on $CD8^+$ T cells with cytotoxic activities and producing IFN-γ (type 1 immune response); (3) such adaptive response seems partially able to control parasitic load, but insufficient, to completely cure the congenital infection; and (4) the cell capacity of congenitally infected neonates to produce IFN-γ determines their parasitic load and the subsequent severity of congenital Chagas disease.

Interpretation attempt of fetal/neonatal immune responses to Trypanosoma cruzi

Neonatal immunity is usually polarized toward a type 2 environment which appears essential for the survival of the fetus. This, in combination with the hypermethylation of the IFN-γ gene in $CD4^+$ T cells, limits the development of type 1 immunity in early life.[132] Thwarting such limitation, as in newborns of *T. cruzi*-infected mothers, requires an activation of fetal/neonatal monocytes and DCs. Monocytes produce IL-12 that synergizes with IL-15 to activate in turn NK cell subpopulations releasing IFN-γ.[133,134] DCs trigger T cell proliferation (preferentially $CD8^+$ T cells) and more IFN-γ release.[119,120] Activation of neonatal DCs can be obtained in the presence of live *T. cruzi* trypomastigotes (as in congenital cases) or, like for monocytes, with soluble molecules released from parasites (e.g., cruzipain, Tc52, HSP70; as in uninfected infants of infected mothers), and antibodies (present in both infant groups; see section: Main biorelevant transferred molecules). The expression of FcR on DC and monocyte membrane can explain the role of antibodies in such activation.

From these observations, it can be postulated that some newborns from *T. cruzi*-infected mothers might naturally auto-cure their congenital infection, either before or at a short time after birth ("unsuccessful" congenital infection in parasitological terms). This could have important consequences both in interpreting neonatal results of laboratory diagnosis, as well as in management of public health control programs related to congenital *T. cruzi* infection (see sections: Laboratory diagnosis of congenital infection with *Trypanosoma cruzi*; Prevention and control of congenital *Trypanosoma cruzi* infection).

Other fetal/neonatal factors susceptible to interfere with the development of congenital Chagas disease

As discussed for mothers, the familial clustering of congenital *T. cruzi* infections (see section: Epidemiological features specific to congenital infection with *Trypanosoma cruzi*) suggests that some neonates might be predisposed to a lower capacity of immune responses, raising the question of a possible role of fetal/neonatal genetic background (different from the maternal one, since it also derives from the father) and/or environmental factors (e.g., malnutrition, poverty, bacterial gut colonization—see section: Other maternal factors involved in transmission of congenital infection) in the susceptibility and outcome of congenital Chagas disease.

Fetal sex is probably not a risk factor for congenital infection, since most studies report similar frequency in both sexes.[19,55,62,135]

It has been shown that living in areas of high vector density during pregnancy (resulting in multiple re-infections with metacyclic trypomasigotes in a previously established chronic infection) is associated with a serious risk of more severe and mortal congenital Chagas disease.[93,136]

Vertical transmission of *Trypanosoma cruzi* in other mammals

Vertical transmission in the natural mammal reservoir of Trypanosoma cruzi

The vertical transmission in mammals involved in the sylvatic cycle of *T. cruzi* (see Chapter 11, Ecological aspects of *Trypanosoma cruzi*: wild hosts and reservoirs) has to be considered as a possible complementary mode, beside vector transmission, of maintaining the parasite reservoir. It does not seem involved in the natural infection of the opossum *Didelphis marsupialis* in endemic areas.[137] Vertical transmission has been observed in some eutherian mammals naturally infected, as dogs, bats,[138–140] and nonhuman primates living in wild conditions or primate centers.[141,142] There is no evidence that chronic maternal *T. cruzi* infection causes fetal loss in nonhuman primates.[143] However, the importance of this vertical mode in the transmission of *T. cruzi* has not been evaluated on a large scale.

Vertical transmission in experimental infection with Trypanosoma cruzi

Guinea pigs were historically the first mammals to be studied for such transmission,[144,145] and one study documents vertical transmission of *T. cruzi* in dogs.[146]

Experiments in mice show acute infection inducing high rates of fetal resorptions and/or pup mortality, in relation to high blood parasite- and TNF-α-levels.[147–150] Indeed, no or rare maternal–fetal transmissions are observed regardless of the time the parasites were inoculated, before or during gestation of mice and rats.[109,147,149,151–160] However, transmission can occur when a blockade of

placental phagocytic activity or placental lesions are induced.[161,162] Two works (by the same team) found transmission being associated with the virulent RA (TcVI)- and not with the CA1 or K98 (TcI)-*T. cruzi* strains.[149,159] Other studies reported high percentages of positive PCR (see section: Detection of neonatal infection (0−4 weeks after birth)) in offspring of mice chronically infected with TcI, TcIV, or TcV, but congenital infection (with live parasites) was not demonstrated in such pups.[163,164] A recent work of our team[110] studied congenital transmission from mouse dams acutely or chronically infected with the *T. cruzi* strains X10 (TcI), Y (TcII), and Tulahuen (TcVI). It showed that congenital infection (detected by blood microscopic examination in offspring submitted to cyclophosphamide-induced immunosuppression in order to activate possible cryptic infection) occurred in approximately 4% of living pups born to dams acutely infected with Y or Tulahuen strains, but not with X10 strain and not in those born to chronically infected animals. Interestingly, this study also shows that (1) parasitemias in transmitting dams are higher than in nontransmitting animals (see section: Parasitic load during pregnancy and transmission of congenital infection); (2) re-inoculation of parasites during gestation in chronically infected mice, strongly increases pup mortality (see section: Other fetal/neonatal factors susceptible to interfere with the development of congenital Chagas disease); and (3) PCR detection of parasitic DNA is unrelated to congenital infection (see section: Detection of neonatal infection (0−4 weeks after birth)). Parasites were not found in rat AF[160] and postnatal transmission by murine milk was not observed[37,160,165] (see section: Possible transmission routes of *Trypanosoma cruzi* parasites). Interestingly, a study showed that the administration of the trypanocidal drug benznidazole to pregnant rats is able to cross the placenta and reach the fetus.[166]

So, despite experimental data confirming some bioclinical observations, mice and rats display lower yield of maternal−fetal parasite transmission than humans and do not bring relevant information on the *T. cruzi*-DTU-congenital infection relationship. Although they have hemochorial placentas like humans, their layer architecture (labyrinthine interdigitation into the murine decidua vs. the villous structure of human placenta; see section: The hematogenous transplacental route: strengths and weaknesses of the trophoblastic barrier) and/or their maternal−fetal blood flow interrelations are different, in addition to their shorter duration of gestation.[38] This makes it difficult to extrapolate data obtained from these animals to the maternal−fetal transmission occurring in human and should encourage further studies in nonhuman higher primates.

Pregnancy outcomes, clinical manifestations, and long-term consequences of congenital Chagas disease

Trypanosoma cruzi *infection and pregnancy outcomes*

The proportions of abortions and stillbirths in women chronically infected with *T. cruzi* were reported to be either similar[167,168] or slightly higher than in uninfected women.[51,60,169−171] Whether they are due to congenital infection and/or placental

dysfunction remains unknown in most cases. However, such records are difficult, and recent and reliable data on abortions or stillbirths in Chagas disease are lacking, which perhaps underestimates rates of maternal−fetal transmission.

Neonatal mortality can occur in untreated severe congenital Chagas disease. We reported mortality rates up to 13% in a Bolivian cohort of congenitally infected newborns studied between 1992 and 1994, while the rate dropped to 2% in another study in 1999−2001, when Bolivia benefited from improved socioeconomic conditions, better maternal care, and extended its vector control programs.[55,93] This latter factor is in line with the role of re-infections in the morbidity and mortality of congenital Chagas disease mentioned in Section, Other fetal/neonatal factors susceptible to interfere with the development of congenital Chagas disease. Neonatal pejorative outcomes are also much higher when acute or reactivated infection (coinfection with HIV) occurs during pregnancy (see sections: Parasitic load during pregnancy and transmission of congenital infection; Other maternal factors involved in transmission of congenital infection).[61,85,86]

Clinical manifestations of congenital Chagas disease

Congenital *T. cruzi* infection, though an acute infection, is frequently asymptomatic at birth, as observed in 40−100% of cases.[55,135,172−177]

Clinical manifestations of congenital Chagas disease can appear within days or weeks after birth, depending on the transplacental transmission time (see section: Timing of maternal−fetal transmission of *Trypanosoma cruzi*). Signs and symptoms that can be observed are non-specific. They are generally similar to those reported in other common congenital infections, due, e.g., to cytomegalovirus and Herpes simplex virus (commonly identified in the acronym TORCH).[178] This and the high frequency of asymptomatic cases highlight the mandatory need for sensitive diagnostic tools to detect such infection close to birth (see section: Laboratory diagnosis of congenital infection with *Trypanosoma cruzi*). *T. cruzi*-infected newborns can exhibit fever, low birth weight (<2500 g), prematurity (gestational age <37 weeks), hepato-splenomegaly, and pneumonitis and more rarely jaundice.[24,49,51,55,62,135,173,175,179−181] The premature rupture of membranes frequently observed in congenital Chagas disease (see section: The hematogenous transplacental route: strengths and weaknesses of the trophoblastic barrier) can result in the birth of premature newborns with immature pulmonary function. Pneumonitis in such cases can be more severe and evolve into respiratory distress syndromes.[55] Growth retardation can be associated with a multisystemic diffusion of parasites in fetus, in addition to being a consequence of placentitis (see section: The hematogenous transplacental route: strengths and weaknesses of the trophoblastic barrier).

More severe clinical manifestations can be also observed in congenital Chagas disease, such as meningoencephalitis (inducing a large range of signs from slight tremors of face or limbs to generalized convulsions) and/or acute myocarditis (resulting in alterations of cardiac rhythm and cardiomegaly),[55,135,179,182−184] particularly in case of maternal coinfection with HIV (see section: Other maternal factors involved in transmission of congenital infection[85,180]).

Purpura and edema (anasarca/fetal hydrops in severe forms) can be also observed.[49,55,185] Anemia and thrombocytopeny have been reported as the main hematological alterations of congenital Chagas disease.[19,49,62,173−175,179,183,185] Megaesophagus or megacolon have been rarely reported in congenital cases.[186−190] Ocular involvement has been also exceptionally mentioned though the possibility of coinfection with *Toxoplasma* has not been eliminated.[179,191] No malformations are detected in such infected newborns.

It is interestingly to note that the oldest clinical reports on congenital Chagas disease from Argentina, Brazil, and Chile indicate the highest morbidity rates, whereas, by contrast, the recent studies in endemic as well as nonendemic countries, more frequently report congenital cases, either asymptomatic or suffering from mild symptoms.

Except in women presenting severe cardiac or digestive forms of Chagas disease before pregnancy,[192,193] gestation generally does not enhance the development of disease in chronically infected women. This allows considering congenital infection and placentitis as being the main short-term consequences of *T. cruzi* infection during pregnancy.

Long-term consequences of congenital infection with Trypanosoma cruzi

Untreated congenital *T. cruzi* infection, whatever the neonatal morbidity, can develop into chronic chagasic myocardiopathy or digestive megaviscera 25−35 years after birth[194] (see Chapter 28, Clinical phases and forms of Chagas disease). However evolution toward severe cardiopathy seems less frequent than in subjects residing in endemic areas and infected by the vector route.[195] This might be related to the maternal imprinting of the fetal/neonatal immune system (mentioned in section: Maternal immunological imprinting/priming of infant immune responses) which can stimulate the immune responses to *T. cruzi*. Untreated cases with meningoencephalitis can suffer severe neurologic sequelae.[196] The transgenerational transmission of parasites (see section: Epidemiological features specific to congenital infection with *Trypanosoma cruzi*) is also a potential long-term consequence of congenital infection.

Laboratory diagnosis of congenital infection with *Trypanosoma cruzi*

The laboratory diagnosis of congenital infection with *T. cruzi* involves, first, the detection of infection in pregnant women, and, second, the confirmation of infection in newborns of positive mothers. Detailed aspects of the laboratory diagnosis of Chagas disease are covered in Chapter 29, Diagnosis of *Trypanosoma cruzi* infection. The present paragraph will focus on the specific aspects of the biological diagnosis of congenital *T. cruzi* infection.

Detection of infection in pregnant women

Positive results to two standard serological tests detecting *T. cruzi*-specific antibodies (indirect hemaglutination, indirect immunofluorescence, or enzyme-linked immunosorbent assay (ELISA)) are necessary for confirming a latent chronic infection in pregnant women as in other patients.[197] Such tests are commercially available at low cost at primary health care level. They have to be performed as soon as pregnancy has been diagnosed, or, if not possible, at any time during pregnancy, including at the time of delivery, or in umbilical cord blood (detecting transferred maternal antibodies).[102] In case of doubtful or discordant results, the tests can be repeated and a third confirmatory test can also be used, such as the radioimmunoprecipitation assays, immunoblot assays using recombinant antigens, and western blot assays using trypomastigote excreted-secreted antigens (TESA-blot).

Sometimes more rapid and simple tests (such as inmunochromatographic, immunodot, immunofiltration tests) are necessary (e.g., when a pregnant woman enter into the maternity just before delivery without previous serodiagnosis, or at primary health care level in rural endemic areas). Positive results obtained with these screening tests need further confirmation with standard serological tests.[102]

However, sensitivity and specificity of serological assays for the diagnosis of Chagas disease appear to be less than previously thought and discordant results can be observed,[198–201] justifying even more the need of using at least two different tests to ensure an accurate diagnosis in pregnant women. If the test results are negative, there is no possible congenital transmission.

In utero detection of fetal infection

As mentioned in Section, Possible transmission routes of *Trypanosoma cruzi* parasites, *T. cruzi* DNA is rarely detected in AF and amniocentesis is currently not recommended for the prenatal diagnosis of *T. cruzi* congenital infection.[27]

Fetal blood sampling (cordocentesis to be performed by experienced clinical practitioners) for standard parasitological or molecular testing has been rarely used for the prenatal diagnosis of congenital *T. cruzi* infections.[202]

Detection of neonatal infection (0–4 weeks after birth)

Blood samples can be collected either at birth, from the umbilical cord (easy to collect without trauma for newborns and mothers) or later in neonates by peripheral venipuncture (from heel, arm, or finger). In case of symptoms suggesting meningoencephalitis, cerebrospinal fluid can also be collected in neonates.

Parasitological tests: Live *T. cruzi* trypomastigotes can be detected in blood samples by microscopic examination of buffy coat from centrifuged heparinized microhematocrit tubes. The latter method is rapid, cheap, affordable, one of both currently recommended gold standards, since detecting around 40 p/mL using four to six tubes (more sensitive than the other parasitological methods, such as microscopic examination of fresh blood samples, fixed blood smears, or thick smear)

and, overall, because detection of parasites in blood definitively confirms congenital infection.[105] The sample has to be examined within 24 h to avoid a decrease of sensitivity due to parasite lysis. Examination of parasites can be done after cutting the tube.[203,204] However, to avoid possible contamination of the examiner, buffy coat can be also examined in the tube without previous rupture using immersion oil[205,206] or by rotating it.[55,122]

The alternative Strout concentration method (so-called microstrout using Eppendorf tubes),[207,208] as well as indirect parasitological methods, such as hemoculture (more expensive and needing weeks before obtaining results), can also be used for detecting blood parasites in congenital *T. cruzi* infection.[55,204] All these parasitological methods, which indisputably confirm infection by showing live parasites, need well-skilled personnel and regular quality controls.

PCR assays: Molecular tests are able to detect low amounts of *T. cruzi* DNA in blood samples of neonates born to infected mothers.[121,204,209–211] *T. cruzi* PCR protocols have been recently compared and efforts have been done to standardize them.[212] Despite of its high sensitivity, PCR does not detect 100% of congenital cases and a negative PCR does not completely exclude a possible congenital infection.

The main question about molecular tests concerns the association between the detection of parasitic DNA and the real presence of live parasites in biological fluids (see section: From maternal–fetal transmission of *Trypanosoma cruzi* to congenital Chagas disease: definitions and limits). As mentioned in section, Main biorelevant transferred molecules, ccfDNA released by *T. cruzi* (dead or live) transferred from mother to uninfected fetuses might be detected by PCR, complicating the interpretation of results, particularly in the case of low intensity amplicons susceptible to correspond to trace amounts of parasitic DNA instead of live parasites. So, an early positive PCR is not absolutely indicative of a congenital infection and even less for its treatment (see section: Treatment of congenital infection with *T. cruzi*). It might be considered that blood sampling later after birth might provide more reliable PCR positive results. However, critical information is still lacking on the stability and duration of parasitic DNA in maternal and umbilical cord blood, and ccfTcDNA might be also produced in some fetuses that have naturally auto-cured their congenital infection (see section: Interpretation attempt of fetal/neonatal immune responses to *Trypanosoma cruzi*).

This probably explains the apparent high maternal–fetal transmission rates reported from human studies considering only results of PCR tests close to birth,[204,209,213,214] as well as in experimental infections,[163,164] whereas reports confirming congenital infection by parasitological or late serological tests display lower rates.[6,110]

Quantitative (real time) PCR that estimates parasite DNA levels, might be useful in completing the results of PCR if protocols are more standardized and a discriminant cut-off value to predict parasite infection is determined.[66,72,82,215,216] New molecular tests targeting exclusively live parasites (e.g., detecting mRNA by reverse transcriptase PCR) or other markers are urgently needed to improve such diagnosis.

Detection of T. cruzi-specific total IgM and IgA antibodies: Antibody isotypes not transferred by mothers are not present in all infected newborns and are also detected in uninfected newborns of infected mothers (see section: Immune responses in newborns of *Trypanosoma cruzi*-infected mothers).[1,101,128,129,217,218] Such detection is presently not recommended for the diagnosis of congenital *T. cruzi* infection.

Detection of IgG/IgM antibodies recognizing Shed Acute Phase Antigen (SAPA): A dot-blot assay detecting IgG antibodies recognizing SAPA has been developed in order to diagnose congenital cases, considering such antibodies as being synthesized by acutely infected fetus/neonates and not transferred from mother.[127] However, further studies indicated that the SAPA-specific antibodies are also detected in chronic patients (including around 50% of infected mothers) and could also be transferred.[219]

A TESA blot (see section: Detection of infection in pregnant women) allows detecting IgM antibodies recognizing a band at 130–200 kDa corresponding to SAPA in blood of congenital cases.[220] The specificity of this TESA (SAPA) blot-IgM is close to 100%, but its sensitivity varies according to the studies.[103,220]

Detection of parasite soluble antigens: Detection of *T. cruzi* soluble antigens in urine and serum by capture ELISA has been proposed for diagnosis of congenital cases. However, these tests did not detect all infected cases.[221,222] Recently, a new test has been developed using nanoparticles to capture, concentrate (by 100-fold), and preserve such antigens in urine (so-called "Chunap"). The antigens are eluted and detected by Western Blot using a monoclonal antibody against *T. cruzi* lipophosphoglycan.[223] This method remains to be validated at a larger scale for the diagnosis of congenital infection.

Placental histopathology: Standard histopathological or immunoenzymatic studies, PCR analyses or in vitro cultures of placental biopsies have been also considered for the diagnosis of congenital *T. cruzi* infection. However, placental parasitism can be either not detected in congenitally infected neonates, or, conversely, observed in uninfected newborns.[22,49,51,70,176,224] So, placental analysis is presently not recommended for the diagnosis of congenital *T. cruzi* infection.

Infant detection of Trypanosoma cruzi *congenital infection (1–12 months after birth)*

If newborns of mothers infected with *T. cruzi* display negative results with the above mentioned tests (mainly parasitological and/or PCR), or if it was not possible to perform such tests, detection of specific antibodies using standard serological assays (as for diagnosis in mothers; see section: Detection of infection in pregnant women) can be carried out in infants when IgG antibodies transferred from the mother have been eliminated. Different studies agree to consider there are no more maternal antibodies 8–9 months after birth.[104,176,225] Such delayed IgG antibody detection is the second currently recommended gold standard method for the diagnosis of congenital infection: a negative serological result indicates the absence of congenital infection, whereas a positive result indicates that the infant is currently

infected. The congenital origin of the *T. cruzi* contamination 8 or more months after birth can be established in areas where possibilities of vector or other transmission routes have been ruled out (i.e., in nonendemic areas or in areas previously endemic but having developed vector and blood bank control programs).[176]

Congenital cases might be also detected at 1–3 months of age with an IgG ELISA using recombinant SAPA, since such maternally transmitted antibodies disappear earlier in babies than conventional antibodies.[218,226] By subtracting the maternal ELISA absorbance values of this test from those in the infant samples, it is possible to detect 90% of congenital cases.[227,228] The beneficial advantage of this simple serological method remains to be validated at a larger scale.

Recommendations

A reliable biological diagnosis of congenital infection is essential to take the decision to initiate a treatment (see section: Treatment of congenital infection with *Trypanosoma cruzi*). So, the criteria defining a "successful" *T. cruzi* congenital infection (to be treated) have to be carefully and clearly specified for public health programs aiming to control such infection (see section: Prevention and control of congenital *Trypanosoma cruzi* infection).[7]

The 2011 conclusions of the WHO technical group on congenital Chagas disease were based on a consensus considering the detection of blood parasites at any time after birth, and/or a positive serology after 8 months of age, as both gold standards for the diagnosis of such infection.[105] An early diagnosis allows a rapid initiation of the treatment and an easier follow-up of the newborns, avoiding their escape from the health system.[229] However, different recent studies have clearly shown a detrimental lack of sensitivity of the parasitological detection.[72,103] Examination of other biological samples of the same neonate week(s) after birth should increase the sensitivity of parasitological detection since neonatal parasitic loads can increase up to 1–3 months after delivery.[72,103,229]

In such a context, what might be the place of PCR for such a diagnosis? Practically, if a PCR is positive in neonates of infected mothers close to birth, it can be considered as alerting the health system to search congenital infection on subsequent samples of these neonates, by using parasitological and/or serological later tests while repeating PCR. This might allow maintaining a more straight contact with the family in order to limit patient escape (see above), and generate a greater efficiency in the management of congenital cases.[230]

Treatment of congenital infection with *Trypanosoma cruzi*

The treatment of *T. cruzi* infection/Chagas disease is reviewed in detail in Chapter 31, Treatment of Chagas disease. The present paragraph will focus on the pediatric application of such treatment. In order to limit the morbidity and mortality of acute infection and prevent the development of further chronic disease at adult

age (see section: Pregnancy outcomes, clinical manifestations, and long-term consequences of congenital Chagas disease), there is a consensus to treat with standard trypanocidal drugs all congenital *T. cruzi* infection detected in newborns or infants, as soon as the diagnosis has been confirmed.[105] Both drugs, benznidazole[55,105,121,175,209,231,232] and nifurtimox,[121,135,175,233,234] can be used to treat congenital cases. Although no comparative and randomized trials have been achieved for congenital infection, it seems that that they have similar curative effects.[121]

The recommended standard doses to be used for both drugs are 10 mg/kg/day (in two to three divided oral subdoses) in neonates and infants till 1 year of age, and 7 mg/kg/day for infants above 1 year, with a 60 day duration of treatment.[105] However, a recent trial using benznidazole at 5 mg/kg in two daily doses for 60 days or at 7.5 mg/kg in a single daily dose for 30 days showed similar efficacy and good tolerance in both regimens, indicating that short treatment should be preferred as it allows reducing its cost.[235]

Such a treatment regimen with doses adapted to increasing body weight is not easy to carry out since pediatric formulations (e.g., liquid formulation) of these drugs remain unavailable. Precautions have to be taken to obtain appropriate dosage since tablets have to be fractionated, crushed, and used as suspension. Full adherence of mothers to scheduled treatment requires a firm relationship between the pediatrician and affected families.[236]

Side effects currently seen in adults (see Chapter 31, Treatment of Chagas disease) are rare, generally mild, and similar in neonates and children treated with benznidazole or nifurtimox.[237,238] The cytogenetic damage and chromosomal aberrations initially reported in chagasic children treated with benznidazole and nifurtimox, respectively,[239] have never been further confirmed. Such low proportion of side effects in neonates efficiently treated with benznidazole is likely related to their lower plasma concentrations of drug (and higher weight-corrected clearance rate) compared to adults.[232]

A negative standard serology is required to confirm cure and this result is generally obtained 3−16 months after the initiation of treatment.[104] The use of PCR/qPCR can help to assess cure (see section: Detection of neonatal infection (0−4 weeks after birth)).[121,209,240] Therapeutic efficacy is around 90−100% in most studies if treatment is applied before 1 year of age.[55,105,121,135,175,176,209]

Prevention and control of congenital *Trypanosoma cruzi* infection

Primary prevention (prophylaxis) of fetal infection with *T. cruzi* aims to prevent infection of pregnant women. This can be obtained by limiting the risk of contamination through vector contacts (see Chapter 10, Control strategies against Triatominae and Chapter 22, Vector transmission: how it works, what transmits, where it occurs) or blood transfusion (see Chapter 24, Other forms of transmission: blood transfusion, organ transplantation, laboratory accidents, oral and sexual transmission). Another strategy which has now clearly shown its efficacy consists of treating infected girls before they enter into their child bearing years[241,242] (see Chapter 31, Treatment of Chagas disease).

Secondary prophylaxis would aim to avoid maternal–fetal parasite transmission from a previously infected pregnant woman using trypanocidal safe drugs. However, the potential teratogenic effects of both currently used trypanocidal drugs, benznidazole and nifurtimox, are not known in humans. Moreover, the side effects of both drugs in adults are hardly acceptable during pregnancy and their curative efficacy is limited in the chronic phase of infection presented by most infected pregnant women (see Chapter 31, Treatment of Chagas disease). For all these reasons, the treatment of *T. cruzi* infection during pregnancy is currently not recommended.[105] Moreover, a recent ex vivo study shows benznidazole and nifurtimox unable to impair parasite invasion of human placental chorionic villi explants and nifurtimox displaying toxic effects on such tissue.[243] However, two other recent reports indicate the use of benznidazole during pregnancy to fight acute or reactivated infections, either because pregnancy was not known[244] or the maternal life was threated.[245] Such treatment prevented congenital transmission with no evidence of side effects either in the mother or the baby. Of course, more studies are necessary to validate these in vivo findings.

So, there is presently an international consensus to consider the best control strategy as associating the detection of congenital infection based on laboratory diagnosis (see section: Laboratory diagnosis of congenital infection with *Trypanosoma cruzi*) with the systematic treatment of positive neonates (see section: Treatment of congenital infection with *Trypanosoma cruzi*).[105] The cost/benefit of this control strategy has been evaluated and shown to be much cheaper than the cumulative costs of managing chagasic patients over years.[246,247] Obviously, its benefit remains fully effective if infants are reintegrated in areas where vector transmission has been controlled. Mathematical models have been proposed to estimate the time period it will take to eliminate congenital transmission in regions where vector transmission was reduced to close to zero.[20] Such a control strategy has been applied successfully in different endemic countries.[175,218,231,248,249] This strategy is presently also recommended in some nonendemic countries, but currently only applied in Spain (Catalonia).[250]

Interestingly, the observation of an association between dwelling in areas of high vector density during pregnancy and the severity of congenital Chagas disease (see section: *Trypanosoma cruzi* infection and pregnancy outcomes)[93] suggests that vector control programs in endemic Latin American areas might unexpectedly contribute to limit the mortality and morbidity of a nonvector-borne infection.

Restriction of breast-feeding in infected mothers has no rational base (see section: Possible transmission routes of *Trypanosoma cruzi* parasites). A recent case report and prospective study of lactating women with Chagas disease treated with benznidazole or nifurtimox showed the limited transference of such drugs into breast milk and the normal clinical evolution of the breastfed babies (with no adverse reactions), suggesting that maternal treatment for Chagas disease during breast feeding is unlikely to present a risk for the breastfed infant.[251–253]

Although out of the scope of this chapter, it is important to mention that cord blood from neonates born to infected mothers should not be used for bone marrow transplantation, due to the risk of inducing an acute CD in an immunosuppressed recipient.[254]

Conclusions

Maternal–fetal transmission of *T. cruzi* can have severe outcomes by compromising survival and/or growth of fetus/neonate, and/or lead to severe clinical forms of chronic infection later in adult life, if infant remains untreated. Congenital *T. cruzi* infection can be found worldwide since such transmission can occur in endemic, as well as nonendemic areas receiving immigrants from endemic regions. Though often asymptomatic at birth and neglected, this congenital infection must be considered as an important public health problem requiring the development of reasonable prevention or control strategies, based on a deep understanding of mechanisms and multiple involved factors. Targeting the group of infected pregnant women at risk of transmission and a reliable diagnosis of neonates displaying a "successful" congenital infection (particularly molecular tests identifying live parasites) are two particularly important points to be improved for a better efficacy of health systems in charge of controlling congenital Chagas disease.

References

1. Carlier Y, Truyens C. Maternal-fetal transmission of *Trypanosoma cruzi*. In: Telleria J, Tibayrenc M, editors. *American trypanosomiasis-Chagas disease. One hundred years of research*. London, Burlington: Elsevier; 2010. p. 539–81.
2. Carlier Y, Truyens C. Congenital Chagas disease as an ecological model of interactions between *Trypanosoma cruzi* parasites, pregnant women, placenta and fetuses. *Acta Trop* 2015;**151**:103–15.
3. Chagas C. Nova entidade morbida do homen. Resumo geral de estudos etiologicos e clinicos. *Mem Inst Oswaldo Cruz* 1911;**3**:219–75.
4. Dao L. Otros casos de la enfermedad de Chagas en el Estado de Guarico (Venezuela): Formas agudas y crónicas; observación sobre la enfermedad de Chagas congénita. *Rev Policlin (Caracas)* 1949;**17**:17–32.
5. World Health Organization. Chagas disease in Latin America: an epidemiological update based on 2010 estimates. *Wkly Epidemiol Rec* 2015;**90**:33–44.
6. Howard EJ, Xiong X, Carlier Y, Sosa-Estani S, Buekens P. Frequency of the congenital transmission of *Trypanosoma cruzi*: a systematic review and meta-analysis. *Brit J Obst Gyn* 2014;**121**:22–33.
7. Carlier Y, Sosa-Estani S, Luquetti AO, Buekens P. Congenital Chagas disease: an update. *Mem Inst Oswaldo Cruz* 2015;**110**:363–8.
8. Luquetti AO, Tavares SB, Siriano Lda R, Oliveira RA, Campos DE, de Morais CA, et al. Congenital transmission of *Trypanosoma cruzi* in central Brazil. A study of 1,211 individuals born to infected mothers. *Mem Inst Oswaldo Cruz* 2015;**110**:369–76.
9. Pellegrino. Migration from Latin America to Europe: trends and policy challenges. *IOM Migration Res* 2004;**16**.
10. Barona-Vilar C, Giménez-Martí MJ, Fraile T, González-Steinbauer C, Parada C, Gil-Brusola A, et al. Prevalence of *Trypanosoma cruzi* infection in pregnant Latin American women and congenital transmission rate in a non-endemic area: the experience of the Valencian Health Programme (Spain). *Epidemiol Infect* 2012;**140**:1896–903.

11. Otero S, Sulleiro E, Molina I, Espiau M, Suy A, Martín-Nalda A, et al. Congenital transmission of *Trypanosoma cruzi* in non-endemic areas: evaluation of a screening program in a tertiary care hospital in Barcelona, Spain. *Am J Trop Med Hyg* 2012;**87**:832−6.
12. Pehrson PO, Wahlgren M, Bengtsson E. Intracranial calcifications probably due to congenital Chagas' disease. *Am J Trop Med Hyg* 1982;**31**:449−51.
13. Jackson Y, Myers C, Diana A, Marti HP, Wolff H, Chappuis F, et al. Congenital transmission of Chagas disease in Latin American immigrants in Switzerland. *Emerg Infect Dis* 2009;**15**:601−3.
14. Lazarte RA, Litman-Mazo F, Crewalk AJ, Keim DE, Baram M, Klassen-Fischer MK, et al. Congenital transmission of Chagas disease—Virginia, 2010. *MMWR Morb Mortal Wkly Rep* 2012;**61**:26.
15. Fearon MA, Scalia V, Huang M, Dines I, Ndao M, Lagacé-Wiens P. A case of vertical transmission of Chagas disease contracted via blood transfusion in Canada. *Can J Infect Dis Med Microbiol* 2013;**24**:32−4.
16. Imai K, Maeda T, Sayama Y, Mikita K, Fujikura Y, Misawa K, et al. Mother-to-child transmission of congenital Chagas disease, Japan. *Emerg Infect Dis* 2014;**20**:146−8.
17. Bittencourt AL. Possible risk factors for vertical transmission of Chagas' disease. *Rev Inst Med Trop Sao Paulo* 1992;**34**:403−8.
18. Schenone H, Gaggero M, Sapunar J, Contreras MC, Rojas A. Congenital Chagas disease of second generation in Santiago, Chile. Report of two cases. *Rev Inst Med Trop Sao Paulo* 2001;**43**:231−2.
19. Sanchez NO, Mora MC, Basombrio MA. High prevalence of congenital *Trypanosoma cruzi* infection and family clustering in Salta, Argentina. *Pediatrics* 2005;**115**:e668−72.
20. Raimundo SM, Massad E, Yang HM. Modelling congenital transmission of Chagas' disease. *Biosystems* 2010;**99**:215−22.
21. Carlier Y. Factors and mechanisms involved in the transmission and development of congenital infection with *Trypanosoma cruzi*. *Rev Soc Bras Med Trop* 2005;**38**(Suppl 2):105−7.
22. Fernandez-Aguilar S, Lambot MA, Torrico F, Alonso-Vega C, Cordoba M, Suarez E, et al. Placental lesions in human *Trypanosoma cruzi* infection. *Rev Soc Bras Med Trop* 2005;**38**(Suppl 2):84−6.
23. Bittencourt AL. Anatomo-pathological aspects of the skin in congenital Chagas' disease. *Rev Inst Med Trop Sao Paulo* 1975;**17**:135−9.
24. Bittencourt AL, Rodrigues de Freitas LA, Galvao de Araujo MO, Jacomo K. Pneumonitis in congenital Chagas' disease. A study of ten cases. *Am J Trop Med Hyg* 1981;**30**:38−42.
25. Soto E, Espinoza J, Nien JK, Kusanovic JP, Erez O, Richani K, et al. Human beta-defensin-2: a natural antimicrobial peptide present in amniotic fluid participates in the host response to microbial invasion of the amniotic cavity. *J Matern Fetal Neonatal Med* 2007;**20**:15−22.
26. Nilo M, Alvarado J, Ramirez M, Espejo E. Hallazgo de tripomastigoto en estudio citoquimico de liqido amniotico. *Parasitologia al dia* 2000;**24**:49−51.
27. Virreira M, Martinez S, Alonso-Vega C, Torrico F, Solano M, Torrico MC, et al. Amniotic fluid is not useful for diagnosis of congenital *Trypanosoma cruzi* infection. *Am J Trop Med Hyg* 2006;**75**:1082−4.
28. Moya PR, Villagra L, Risco J. Congenital Chagas disease: anatomopathological findings in the placenta and umbilical cord. *Rev Fac Cienc Med Cordoba* 1979;**37**:21−7.
29. Medina-Lopes MD. Transmission of *Trypanosoma cruzi* in a case, during lactation, in a non-endemic area. *Rev Soc Bras Med Trop* 1988;**21**:151−3.

30. Mazza S, Montana A, Benitez C, Janzi E. Transmision del *Schizotrypanum cruzi* al nino por leche de la madre con enfermedad de Chagas. Pubicaciones Mision de Estudios de Patologia Regional Argentina (MEPRA). *Universidad de Buenos-Aires* 1936;**28**:41–6.
31. Jörg ME. The transmission of *Trypanosoma cruzi* via human milk. *Rev Soc Bras Med Trop* 1992;**25**:83.
32. Bittencourt AL, Sadigursky M, Da Silva AA, Menezes CA, Marianetti MM, Guerra SC, et al. Evaluation of Chagas' disease transmission through breast-feeding. *Mem Inst Oswaldo Cruz* 1988;**83**:37–9.
33. Amato NV, Matsubara L, Campos R, Moreira AA, Pinto PL, Facciol R, et al. *Trypanosoma cruzi* in the milk of women with chronic Chagas disease. *Rev Hosp Clin Fac Med Sao Paulo* 1992;**47**:10–11.
34. Norman FF, López-Vélez R. Chagas disease and breast-feeding. *Emerg Inf Dis* 2013;**19**:1561–6.
35. Hoft DF. Differential mucosal infectivity of different life stages of *Trypanosoma cruzi*. *Am J Trop Med Hyg* 1996;**55**:360–4.
36. Cortez C, Yoshida N, Bahia D, Sobreira TJ. Structural basis of the interaction of a *Trypanosoma cruzi* surface molecule implicated in oral infection with host cells and gastric mucin. *PLoS One* 2012;**7**:e42153.
37. Martins LP, Castanho RE, Nogueira AB, Silva OT, Gusmão AS. Incidence of *Trypanosoma cruzi* transmission through breastfeeding during acute experimental Chagas disease. *Braz J Infect Dis* 2011;**15**:116–18.
38. Benirschke K, Kaufmann P, Baergen R. *Pathology of the human placenta*. New York: Springer Science + Business Media; 2006.
39. Zeldovich VB, Clausen CH, Bradford E, Fletcher DA, Maltepe E, Robbins JR, et al. Placental syncytium forms a biophysical barrier against pathogen invasion. *PLoS Pathog* 2013;**9**:e1003821.
40. Koga K, Izumi G, Mor G, Fujii T, Osuga Y. Toll-like receptors at the maternal-fetal interface in normal pregnancy and pregnancy complications. *Am J Reprod Immunol* 2014;**72**:192–205.
41. Nanaev AK, Kosanke G, Kemp B, Frank HG, Huppertz B, Kaufmann P. The human placenta is encircled by a ring of smooth muscle cells. *Placenta* 2000;**21**:122–5.
42. Frank F, Sartori MJ, Asteggiano C, Lin S, de Fabro SP, Fretes RE. The effect of placental subfractions on *Trypanosoma cruzi*. *Exp Mol Pathol* 2000;**69**:144–51.
43. Díaz-Luján C, Triquell MF, Schijman A, Paglini P, Fretes RE. Differential susceptibility of isolated human trophoblasts to infection by *Trypanosoma cruzi*. *Placenta* 2012;**33**:264–70.
44. Liempi A, Castillo C, Duaso J, Droguett D, Sandoval A, Barahona K, et al. *Trypanosoma cruzi* induces trophoblast differentiation: a potential local antiparasitic mechanism of the human placenta? *Placenta* 2014;**35**:1035–42.
45. Sartori MJ, Lin S, Frank FM, Malchiodi EL, de Fabro SP. Role of placental alkaline phosphatase in the interaction between human placental trophoblast and *Trypanosoma cruzi*. *Exp Mol Pathol* 2002;**72**:84–90.
46. Duaso J, Rojo G, Cabrera G, Galanti N, Bosco C, Maya JD, et al. *Trypanosoma cruzi* induces tissue disorganization and destruction of chorionic villi in an ex vivo infection model of human placenta. *Placenta* 2010;**31**:705–11.
47. Castillo C, Ramírez G, Valck C, Aguilar L, Maldonado I, Rosas C, et al. The interaction of classical complement component C1 with parasite and host calreticulin mediates *Trypanosoma cruzi* infection of human placenta. *PLoS Negl Trop Dis* 2013;**7**:e2376.

48. Bittencourt AL. Chagasic placentitis and congenital transmission of Chagas' disease. *Rev Inst Med Trop Sao Paulo* 1963;**5**:62–7.
49. Bittencourt AL. Congenital Chagas disease. *Am J Dis Child* 1976;**130**:97–103.
50. Altemani AM, Bittencourt AL, Lana AM. Immunohistochemical characterization of the inflammatory infiltrate in placental Chagas disease: a qualitative and quantitative analysis. *Am J Trop Med Hyg* 2000;**62**:319–24.
51. Azogue E, La Fuente C, Darras C. Congenital Chagas' disease in Bolivia: epidemiological aspects and pathological findings. *Trans R Soc Trop Med Hyg* 1985;**79**:176–80.
52. Haider S, Knofler M. Human tumour necrosis factor: physiological and pathological roles in placenta and endometrium. *Placenta* 2009;**30**:111–23.
53. Myatt L, Cui X. Oxidative stress in the placenta. *Histochem Cell Biol* 2004;**122**: 369–82.
54. Kurup SP, Tarleton RL. Perpetual expression of PAMPs necessary for optimal immune control and clearance of a persistent pathogen. *Nat Commun* 2013;**4**:2616.
55. Torrico F, Alonso-Vega C, Suarez E, Rodriguez P, Torrico MC, Dramaix M, et al. Maternal *Trypanosoma cruzi* infection, pregnancy outcome, morbidity, and mortality of congenitally infected and non-infected newborns in Bolivia. *Am J Trop Med Hyg* 2004;**70**:201–9.
56. Biggar RJ, Lee TH, Wen L, Broadhead R, Kumwenda N, Taha TE, et al. The role of transplacental microtransfusions of maternal lymphocytes in HIV transmission to newborns. *AIDS* 2008;**22**:2251–6.
57. Aagaard K, Ma J, Antony KM, Ganu R, Petrosino J, Versalovic J. The placenta harbors a unique microbiome. *Sci Transl Med* 2014;**6**:237ra65.
58. Juiz NA, Cayo NM, Burgos M, Salvo ME, Nasser JR, Búa J, et al. Human polymorphisms in placentally expressed genes and their association with susceptibility to congenital *Trypanosoma cruzi* infection. *J Infect Dis* 2016;**213**:1299–306.
59. Jauniaux E, Gulbis B, Burton GJ. Physiological implications of the materno-fetal oxygen gradient in human early pregnancy. *Reprod Biomed Online* 2003;**7**:250–3.
60. Bittencourt AL, Barbosa HS. Incidence of congenital transmission of Chagas' disease in abortion. *Rev Inst Med Trop Sao Paulo* 1972;**14**:257–9.
61. Moretti E, Basso B, Castro I, Carrizo P, Chaul M, Barbieri G, et al. Chagas' disease: study of congenital transmission in cases of acute maternal infection. *Rev Soc Bras Med Trop* 2005;**38**:53–5.
62. Salas NA, Cot M, Schneider D, Mendoza B, Santalla JA, Postigo J, et al. Risk factors and consequences of congenital Chagas disease in Yacuiba, south Bolivia. *Trop Med Int Health* 2007;**12**:1498–505.
63. Zingales B, Miles MA, Campbell DA, Tibayrenc M, Macedo AM, Teixeira MM, et al. The revised *Trypanosoma cruzi* subspecific nomenclature: rationale, epidemiological relevance and research applications. *Infect Genet Evol* 2012;**12**:240–53.
64. Barnabé C, Mobarec HI, Jurado MR, Cortez JA, Brenière SF. Reconsideration of the seven discrete typing units within the species *Trypanosoma cruzi*, a new proposal of three reliable mitochondrial clades. *Infect Genet Evol* 2016;**39**:176–86.
65. Virreira M, Alonso-Vega C, Solano M, Jijena J, Brutus L, Bustamante Z, et al. Congenital Chagas disease in Bolivia is not associated with DNA polymorphism of *Trypanosoma cruzi*. *Am J Trop Med Hyg* 2006;**75**:871–9.
66. Virreira M, Truyens C, Alonso-Vega C, Brutus L, Jijena J, Torrico F, et al. Comparison of *Trypanosoma cruzi* lineages and levels of parasitic DNA in infected mothers and their newborns. *Am J Trop Med Hyg* 2007;**77**:102–6.

67. Burgos JM, Altcheh J, Bisio M, Duffy T, Valadares HM, Seidenstein ME, et al. Direct molecular profiling of minicircle signatures and lineages of *Trypanosoma cruzi* bloodstream populations causing congenital Chagas disease. *Int J Parasitol* 2007;**37**: 1319–27.
68. Corrales RM, Mora MC, Negrette OS, Diosque P, Lacunza D, Virreira M, et al. Congenital Chagas disease involves *Trypanosoma cruzi* sub-lineage IId in the northwestern province of Salta, Argentina. *Infect Genet Evol* 2009;**9**:278–82.
69. Diez C, Lorenz V, Ortiz S, Gonzalez V, Racca A, Bontempi I, et al. Genotyping of *Trypanosoma cruzi* sublineage in human samples from a North-East Argentina area by hybridization with DNA probes and specific polymerase chain reaction (PCR). *Am J Trop Med Hyg* 2010;**82**:67–73.
70. Bisio M, Seidenstein ME, Burgos JM, Ballering G, Risso M, Pontoriero R, et al. Urbanization of congenital transmission of *Trypanosoma cruzi*: prospective polymerase chain reaction study in pregnancy. *Trans R Soc Trop Med Hyg* 2011;**105**:543–9.
71. Ortiz S, Zulantay I, Solari A, Bisio M, Schijman A, Carlier Y, et al. Presence of *Trypanosoma cruzi* in pregnant women and typing of lineages in congenital cases. *Acta Trop* 2012;**124**:243–6.
72. Bua J, Volta BJ, Perrone AE, Scollo K, Velazquez EB, Ruiz AM, et al. How to improve the early diagnosis of *Trypanosoma cruzi* infection: relationship between validated conventional diagnosis and quantitative DNA amplification in congenitally infected children. *PLoS Negl Trop Dis* 2013;**10**:e2476.
73. Garcia A, Ortiz S, Iribarren C, Bahamonde MI, Solari A. Congenital co-infection with different *Trypanosoma cruzi* lineages. *Parasitol Int* 2014;**63**:138–9.
74. Llewellyn MS, Messenger LA, Luquetti AO, Garcia L, Torrico F, Tavares SB, et al. Deep sequencing of the *Trypanosoma cruzi* GP63 surface proteases reveals diversity and diversifying selection among chronic and congenital Chagas disease patients. *PLoS Negl Trop Dis* 2015;**9**:e0003458.
75. Bittencourt AL, Mota E, Povoa M. Isoenzyme characterization of *Trypanosoma cruzi* from congenital cases of Chagas' disease. *Ann Trop Med Parasitol* 1985;**79**:393–6.
76. del Puerto F, Sanchez Z, Nara E, Meza G, Paredes B, Ferreira E, et al. *Trypanosoma cruzi* lineages detected in congenitally infected infants and *Triatoma infestans* from the same disease-endemic region under entomologic surveillance in Paraguay. *Am J Trop Med Hyg* 2010;**82**:386–90.
77. Pavia PX, Montilla M, Florez C, Herrera G, Ospina JM, Manrique F, et al. The first case of congenital Chagas' disease analyzed by AP-PCR in Colombia. *Biomedica* 2009;**29**:513–22.
78. Brutus L, Ernould JC, Postigo J, Romero M, Schneider D, Santalla JA. Influence of pregnancy on *Trypanosoma cruzi* parasitemia in chronically infected women in a rural Bolivian community. *Am J Trop Med Hyg* 2011;**84**:808–12.
79. Siriano L, da R, Luquetti AO, Boaventura Avelar J, Marra NL, de Castro AM. Chagas disease: increased parasitemia during pregnancy detected by hemoculture. *Am J Trop Med Hyg* 2011;**84**:569–74.
80. Hermann E, Truyens C, Alonso-Vega C, Rodriguez P, Berthe A, Torrico F, et al. Congenital transmission of *Trypanosoma cruzi* is associated with maternal enhanced parasitemia and decreased production of interferon-gamma in response to parasite antigens. *J Infect Dis* 2004;**189**:1274–81.
81. Brutus L, Castillo H, Bernal C, Salas NA, Schneider D, Santalla JA, et al. Detectable *Trypanosoma cruzi* parasitemia during pregnancy and delivery as a risk factor for congenital Chagas disease. *Am J Trop Med Hyg* 2010;**83**:1044–7.

82. Bua J, Volta BJ, Velazquez EB, Ruiz AM, Rissio AM, Cardoni RL. Vertical transmission of *Trypanosoma cruzi* infection: quantification of parasite burden in mothers and their children by parasite DNA amplification. *Trans R Soc Trop Med Hyg* 2012;**106**:623–8.
83. Kaplinski M, Jois M, Galdos-Cardenas G, Rendell VR, Shah V, Do RQ, et al. Sustained domestic vector exposure is associated with increased Chagas cardiomyopathy risk but decreased parasitemia and congenital transmission risk among young women in Bolivia. *Clin Infect Dis* 2015;61918–26.
84. Rendell VR, Gilman RH, Valencia E, Galdos-Cardenas G, Verastegui M, Sanchez L, et al. *Trypanosoma cruzi*-infected pregnant women without vector exposure have higher parasitemia levels: implications for congenital transmission risk. *PLoS One* 2015;**10**: e0119527.
85. Freilij H, Altcheh J, Muchinik G. Perinatal human immunodeficiency virus infection and congenital Chagas' disease. *Pediatr Infect Dis* 1995;**14**:161–2.
86. Scapellato PG, Bottaro EG, Rodriguez-Brieschke MT. Mother-child transmission of Chagas disease: could coinfection with human immunodeficiency virus increase the risk? *Rev Soc Bras Med Trop* 2009;**42**:107–9.
87. Cardoni RL, Garcia MM, De Rissio AM. pro-inflammatory and anti-inflammatory cytokines in pregnant women chronically infected with *Trypanosoma cruzi*. *Acta Trop* 2004;**90**:65–72.
88. Garcia MM, de Rissio AM, Villalonga X, Mengoni E, Cardoni RL. Soluble tumor necrosis factor (TNF) receptors (sTNF-R1 and -R2) in pregnant women chronically infected with *Trypanosoma cruzi* and their children. *Am J Trop Med Hyg* 2008;**78**:499–503.
89. Vekemans J, Truyens C, Torrico F, Solano M, Torrico MC, Rodrigue P, et al. Maternal *Trypanosoma cruzi* infection upregulates capacity of uninfected neonate cells to produce pro- and anti-inflammatory cytokines. *Infect Immun* 2000; **68**:5430–4.
90. Cuna WR, Choque AG, Passera R, Rodriguez C. Pro-inflammatory cytokine production in chagasic mothers and their uninfected newborns. *J Parasitol* 2009;**95**:891–4.
91. Racicot K, Kwon JY, Aldo P, Silasi M, Mor G. Understanding the complexity of the immune system during pregnancy. *Am J Reprod Immunol* 2014;**72**:107–16.
92. Azogue E. Women and congenital Chagas' disease in Santa Cruz, Bolivia: epidemiological and sociocultural aspects. *Soc Sci Med* 1993;**37**:503–11.
93. Torrico F, Alonso-Vega C, Suarez E, Tellez T, Brutus L, Rodriguez P, et al. Are maternal re-infections with *Trypanosoma cruzi* associated with higher morbidity and mortality of congenital Chagas disease?. *Trop Med Int Health* 2006;**11**:628–35.
94. Rytter MJ, Kolte L, Briend A, Friis H, Christensen VB. The immune system in children with malnutrition—a systematic review. *PLoS One* 2014;**9**:e105017.
95. Dolcini GL, Solana ME, Andreani G, Celentano AM, Parodi LM, Donato AM, et al. *Trypanosoma cruzi* (Chagas' disease agent) reduces HIV-1 replication in human placenta. *Retrovirology* 2008;**5**:53.
96. Romano-Keeler J, Weitkamp JH. Maternal influences on fetal microbial colonization and immune development. *Pediatr Res* 2015;**77**:189–95.
97. Galili U. Anti-Gal: an abundant human natural antibody of multiple pathogeneses and clinical benefits. *Immunology* 2013;**140**:1–11.
98. Simister NE. Placental transport of immunoglobulin G. *Vaccine* 2003;**21**:3365–9.
99. Miles MA, Castro C, Macedo V, Draper CC. Letter: *Trypanosoma cruzi*—prenatal transfer of maternal antibody in man. *Trans R Soc Trop Med Hyg* 1975;**69**:286.

100. Breniere SF, Bailly M, Carrasco R, Carlier Y. Transmission transplacentaire des anticorps anti-*Trypanosoma cruzi*. *Cah ORSTOM Ser Ent Med et Parasitol* 1983;**21**: 139–40.
101. Truyens C, Hermann E, Alonso-Vega C, Rodriguez P, Vekemans J, Torrico F, et al. Immune responses of non-infected neonates of mothers infected with *Trypanosoma cruzi*. *Rev Soc Bras Med Trop* 2005;**38**(Suppl 2):96–100.
102. Sosa-Estani S, Gamboa-Leon MR, Cid-Lemus J, Althabe F, Alger J, Almendares O, et al. Use of a rapid test on umbilical cord blood to screen for *Trypanosoma cruzi* infection in pregnant women in Argentina, Bolivia, Honduras, and Mexico. *Am J Trop Med Hyg* 2008;**79**:755–9.
103. Bern C, Verastegui M, Gilman RH, Lafuente C, Galdos-Cardenas G, Calderon M, et al. Congenital *Trypanosoma cruzi* transmission in Santa Cruz, Bolivia. *Clin Infect Dis* 2009;**49**:1667–74.
104. Chippaux JP, Clavijo AN, Santalla JA, Postigo JR, Schneider D, Brutus L. Antibody drop in newborns congenitally infected by *Trypanosoma cruzi* treated with benznidazole. *Trop Med Int Health* 2010;**15**:87–93.
105. Carlier Y, Torrico F, Sosa-Estani S, Russomando G, Luquetti L, Freilij H, et al. Congenital Chagas disease: recommendations for diagnosis, treatment and control of newborns, siblings and pregnant women. *PLoS Negl Trop Dis* 2011;**5**:e1250.
106. Neves SF, Eloi-Santos S, Ramos R, Rigueirinho S, Gazzinelli G, Correa-Oliveira R. In utero sensitization in Chagas' disease leads to altered lymphocyte phenotypic patterns in the newborn cord blood mononuclear cells. *Parasite Immunol* 1999;**21**:631–9.
107. Rivera MT, Marques de Araujo S, Lucas R, Deman J, Truyens C, Defresne MP, et al. High tumor necrosis factor alpha (TNF-alpha) production in *Trypanosoma cruzi*-infected pregnant mice and increased TNF-alpha gene transcription in their offspring. *Infect Immun* 1995;**63**:591–5.
108. Didoli GL, Davila HO, Feldman S, di Masso R, Revelli SS, Bottasso OA. Protected *Trypanosoma cruzi* infection in rats born to mothers receiving interferon-gamma during gestation is associated with a decreased intramacrophage parasite growth and preferential synthesis of specific IgG2b antibodies. *Int J Immunopharmacol* 2000;**22**:45–55.
109. Alarcón M, Pérez MC, Villarreal J, Araujo S, Goncalves L, González A, et al. Detection of *Trypanosoma cruzi* DNA in the placenta and fetuses of mice with Chagasic acute infection. *Invest Clin* 2009;**50**:335–45.
110. Cencig S, Coltel N, Truyens C, Carlier Y. Fertility, gestation outcome and parasite congenital transmissibility in mice infected with TcI, TcII and TcVI genotypes of *Trypanosoma cruzi*. *PLoS Negl Trop Dis* 2013;**7**:e2271.
111. Garcia-Silva MR, Cabrera-Cabrera F, das Neves RF, Souto-Padrón T, de Souza W, Cayota A. Gene expression changes induced by *Trypanosoma cruzi* shed microvesicles in mammalian host cells: relevance of tRNA-derived halves. *Biomed Res Int* 2014;**2014**:305239.
112. Maloney S, Smith A, Furst DE, Myerson D, Rupert K, Evans PC, et al. Microchimerism of maternal origin persists into adult life. *J Clin Invest* 1999;**104**:41–7.
113. Jonsson AM, Uzunel M, Götherström C, Papadogiannakis N, Westgren M. Maternal microchimerism in human fetal tissues. *Am J Obstet Gynecol* 2008;**198**:325.e1–6.
114. AbdelHalim RM, Ramadan DI, Zeyada R, Nasr AS, Mandour IA. Circulating maternal total cell-free DNA, cell-free fetal DNA and soluble endoglin levels in preeclampsia: predictors of adverse fetal outcome? A cohort study. *Mol Diagn Ther* 2016;**2**:135–49.

115. Jonakait GM. The effects of maternal inflammation on neuronal development: possible mechanisms. *Int J Dev Neurosci* 2007;**25**:415–25.
116. Carlier Y, Truyens C. Influence of maternal infection on offspring resistance towards parasites. *Parasitol Today* 1995;**11**:94–9.
117. Dauby N, Goetghebuer T, Kollmann TR, Levy J, Marchant A. Uninfected but not unaffected: chronic maternal infections during pregnancy, fetal immunity, and susceptibility to postnatal infections. *Lancet Infect Dis* 2012;**12**:330–40.
118. Dauby N, Alonso-Vega C, Suarez E, Flores A, Hermann E, Cordova M, et al. Maternal infection with *Trypanosoma cruzi* and congenital Chagas disease induce a trend to a type 1 polarization of infant immune responses to vaccines. *PLoS Neg Trop Dis* 2009;**3**:e571.
119. Rodriguez P, Carlier Y, Truyens C. Activation of cord blood myeloid dendritic cells by *Trypanosoma cruzi* and parasite-specific antibodies, proliferation of CD8+ T cells, and production of IFN-γ. *Med Microbiol Immunol* 2012;**201**:157–69.
120. Rodriguez P, Carlier Y, Truyens C. *Trypanosoma cruzi* activates cord blood myeloid dendritic cells independently of cell infection. *Med Microbiol Immunol* 2012;**201**:287–96.
121. Schijman AG, Altcheh J, Burgos JM, Biancardi M, Bisio M, Levin MJ, et al. Aetiological treatment of congenital Chagas' disease diagnosed and monitored by the polymerase chain reaction. *J Antimicrob Chemother* 2003;**52**:441–9.
122. Torrico MC, Solano M, Guzman JM, Parrado R, Suarez E, Alonzo-Vega C, et al. Estimation of the parasitemia in *Trypanosoma cruzi* human infection: high parasitemias are associated with severe and fatal congenital Chagas disease. *Rev Soc Bras Med Trop* 2005;**38**(Suppl 2):58–61.
123. Mayer JP, Biancardi M, Altcheh J, Freilij H, Weinke T, Liesenfeld O. Congenital infections with *Trypanosoma cruzi* or *Toxoplasma gondii* are associated with decreased serum concentrations of interferon-gamma and interleukin-18 but increased concentrations of interleukin-10. *Ann Trop Med Parasitol* 2010;**104**:485–92.
124. Fernández-Villegas A, Thomas MC, Carrilero B, Téllez C, Marañón C, Murcia L, et al. The innate immune response status correlates with a divergent clinical course in congenital Chagas disease of twins born in a non-endemic country. *Acta Trop* 2014;**140**:84–90.
125. Hermann E, Alonso-Vega C, Berthe A, Truyens C, Flores A, Cordova M, et al. Human congenital infection with *Trypanosoma cruzi* induces phenotypic and functional modifications of cord blood NK cells. *Pediatr Res* 2006;**60**:38–43.
126. Hermann E, Truyens C, Alonso-Vega C, Even J, Rodriguez P, Berthe A, et al. Human fetuses are able to mount an adultlike CD8T-cell response. *Blood* 2002;**100**:2153–8.
127. Reyes MB, Lorca M, Munoz P, Frasch AC. Fetal IgG specificities against *Trypanosoma cruzi* antigens in infected newborns. *Proc Natl Acad Sci USA* 1990;**87**:2846–50.
128. Di Pentima MC, Edwards M. Enzyme-linked immunosorbent assay for IgA antibodies to *Trypanosoma cruzi* in congenital infection. *Am J Trop Med Hyg* 1999;**60**:211–14.
129. Rodriguez P, Truyens C, Alonso-Vega C, Flores A, Cordova M, Suarez E, et al. Serum levels of IgM and IgA antibodies to anti-*Trypanosoma cruzi* in samples of blood from newborns from mothers with positive serology for Chagas disease. *Rev Soc Bras Med Trop* 2005;**38**(Suppl 2):62–4.
130. Laucella SA, Postan M, Martin D, Hubby Fralish B, Albareda MC, Alvarez MG, et al. Frequency of interferon-gamma-producing T cells specific for *Trypanosoma cruzi*

inversely correlates with disease severity in chronic human Chagas disease. *J Infect Dis* 2004;**189**:909–18.

131. Eloi-Santos SM, Novato-Silva E, Maselli VM, Gazzinelli G, Colley DG, Correa-Oliveira R. Idiotypic sensitization in utero of children born to mothers with schistosomiasis or Chagas' disease. *J Clin Invest* 1989;**84**:1028–31.
132. Prabhudas M, Adkins B, Gans H, King C, Levy O, Ramilo O, et al. Challenges in infant immunity: implications for responses to infection and vaccines. *Nat Immunol* 2011;**12**:189–94.
133. Guilmot A, Bosse J, Carlier Y, Truyens C. Monocytes play an IL-12-dependent crucial role in driving cord blood NK cells to produce IFN-g in response to *Trypanosoma cruzi*. *PLoS Negl Trop Dis* 2013;**7**:e2291.
134. Guilmot A, Carlier Y, Truyens C. Differential IFN-γ production by adult and neonatal blood CD56+ natural killer (NK) and NK-like-T cells in response to *Trypanosoma cruzi* and IL-15. *Parasite Immunol* 2014;**36**:43–52.
135. Freilij H, Altcheh J. Congenital Chagas' disease: diagnostic and clinical aspects. *Clin Infect Dis* 1995;**21**:551–5.
136. Brutus L, Schneider D, Postigo J, Romero M, Santalla J, Chippaux JP. Congenital Chagas disease: diagnostic and clinical aspects in an area without vectorial transmission, Bermejo, Bolivia. *Acta Trop* 2008;**106**:195–9.
137. Jansen AM, Madeira FB, Deane MP. *Trypanosoma cruzi* infection in the opossum *Didelphis marsupialis*: absence of neonatal transmission and protection by maternal antibodies in experimental infections. *Mem Inst Oswaldo Cruz* 1994;**89**:41–5.
138. Villela EA. A transmissao intra-uterina da moléstia de Chagas. Encefalite congênita pelo *Trypanosoma cruzi* (Nota prévia). *Folia Méd* 1923;**4**:41–3.
139. Barr SC, Van Beek O, Carlisle-Nowak MS, Lopez JW, Kirchhoff LV, Allison N, et al. *Trypanosoma cruzi* infection in Walker hounds from Virginia. *Am J Vet Res* 1995;**56**:1037–44.
140. Añez N, Crisante G, Soriano PJ. *Trypanosoma cruzi* congenital transmission in wild bats. *Acta Trop* 2009;**109**:78–80.
141. Dorn PL, Daigle ME, Combe CL, Tate AH, Stevens L, Phillippi-Falkenstein KM. Low prevalence of Chagas parasite infection in a nonhuman primate colony in Louisiana. *J Am Assoc Lab Anim Sci* 2012;**51**:443–7.
142. Minuzzi-Souza TT, Nitz N, Knox MB, Reis F, Hagström L, Cuba CA, et al. Vector-borne transmission of Trypanosoma cruzi among captive Neotropical primates in a Brazilian zoo. *Parasit Vectors* 2016;**9**:39.
143. Grieves JL, Hubbard GB, Williams JT, Vandeberg JL, Dick Jr EJ, Lopez-Alvarenga JC, et al. *Trypanosoma cruzi* in non-human primates with a history of stillbirths: a retrospective study (*Papio hamadryas* spp.) and case report (*Macaca fascicularis*). *J Med Primatol* 2008;**37**:318–28.
144. Mayer M, Rocha-Lima H. Zum verhalten von *Schizotrypanum cruzi* in warm blutern und arthropoden. *Arch FU Schiffs-u Tropenhyg* 1914;**18**:257–92.
145. Nattan-Larrier L. Hérédité des infections expérimentales à *Schizotrypanum cruzi*. *Bull Soc Pathol Exot* 1921;**14**:232–8.
146. Rodríguez-Morales O, Ballinas-Verdugo MA, Alejandre-Aguilar R, Reyes PA, Arce-Fonseca M. *Trypanosoma cruzi* connatal transmission in dogs with Chagas disease: experimental case report. *Vector Borne Zoonotic Dis* 2011;**11**:1365–70.
147. Mjihdi A, Lambot MA, Stewart IJ, Detournay O, Noel JC, Carlier Y, et al. Acute *Trypanosoma cruzi* infection in mouse induces infertility or placental parasite invasion

and ischemic necrosis associated with massive fetal loss. *Am J Pathol* 2002;**161**: 673–80.

148. Mjihdi A, Truyens C, Detournay O, Carlier Y. Systemic and placental productions of tumor necrosis factor contribute to induce fetal mortality in mice acutely infected with *Trypanosoma cruzi*. *Exp Parasitol* 2004;**107**:58–64.
149. Solana ME, Celentano AM, Tekiel V, Jones M, Gonzalez Cappa SM. *Trypanosoma cruzi*: effect of parasite subpopulation on murine pregnancy outcome. *J Parasitol* 2002; **88**:102–6.
150. Solana ME, Alba Soto CD, Fernandez MC, Poncini CV, Postan M, Gonzalez Cappa SM. Reduction of parasite levels in blood improves pregnancy outcome during experimental *Trypanosoma cruzi* infection. *Parasitology* 2009;**136**:627–39.
151. Apt W, Naquira C, Strozzi L. Congenital transmission of trypanosomiasis cruzi. III. In mice with acute and chronic infection. *Bol Chil Parasitol* 1968;**23**:15–19.
152. Apt W, Naquira C, Tejada A, Strozzi L. Congenital transmission of *Trypanosoma cruzi*. II. In rats with acute and chronic infections. *Bol Chil Parasitol* 1968;**23**:9–15.
153. Werner H, Egger I. Congenital chagas—influence of *Trypanosoma cruzi* infection on embryonic development in pregnant mice. *Z Tropenmed Parasitol* 1971;**22**:224–34.
154. Cabeza MP, Chambo GJ, Laguens RP. Congenital disease secondary to the chronic infection of mice with *Trypanosoma cruzi*. Experimental model of congenital Chagas disease. *Medicina (B Aires)* 1980;**40**(Suppl 1):40–4.
155. de Cunio RW, Olmos JA, de Mercau TN, Blanca RL, de Marteau DF, Ontivero MI, et al. Experimental congenital Chagas-Mazza disease. *Medicina (B Aires)* 1980;**40** (Suppl 1):50–5.
156. Andrade S. The influence of the strain of Trypanosoma cruzi in placental infections in mice. *Trans R Soc Trop Med Hyg* 1982;**76**:123–8.
157. Carlier Y, Rivera MT, Truyens C, Puissant F, Milaire J. Interactions between chronic murine Trypanosoma cruzi infection and pregnancy: fetal growth retardation. *Am J Trop Med Hyg* 1987;**37**:534–40.
158. Davila HO, Revelli SS, Moreno HS, Valenti JL, Musso OC, Poli HO, et al. Infection with *Trypanosoma cruzi* during pregnancy in rats and a decrease in chronic myocardial lesions in their infected offspring. *Am J Trop Med Hyg* 1994;**50**:506–11.
159. Gonzalez Cappa SM, Mirkin GA, Solana ME, Tekiel VS. *Trypanosoma cruzi* pathology. Strain dependent? *Medicina (B Aires)* 1999;**59**(Suppl 2):69–74.
160. Moreno EA, Rivera IM, Moreno SC, Alarcon ME, Lugo-Yarbuh A. Vertical transmission of *Trypanosoma cruzi* in Wistar rats during the acute phase of infection. *Invest Clin* 2003;**44**:241–54.
161. Werner H, Kunert H. Causes of congenital Protozoa infection. *Z Tropenmed Parasitol* 1958;**9**:17–27.
162. Delgado MA, Santos-Buch CA. Transplacental transmission and fetal parasitosis of *Trypanosoma cruzi* in outbred white Swiss mice. *Am J Trop Med Hyg* 1978;**27**: 1108–15.
163. Hall CA, Pierce EM, Wimsatt AN, Hobby-Dolbeer T, Meers JB. Virulence and vertical transmission of two genotypically and geographically diverse isolates of *Trypanosoma cruzi* in mice. *J Parasitol* 2010;**96**:371–6.
164. Alkmim-Oliveira SM, Costa-Martins AG, Kappel HB, Correia D, Ramirez LE, Lages-Silva E. *Trypanosoma cruzi* experimental congenital transmission associated with TcV and TcI subpatent maternal parasitemia. *Parasitol Res* 2013;**112**:671–8.

165. Davila HO, Revelli S, Moreno HS, Valenti JL, Musso OC, Poli HO, et al. Attenuated *Trypanosoma cruzi* infection in young rats nursed on infected mothers undergoing interferon-gamma treatment during pregnancy. *Immunopharmacology* 1997;**37**:1–6.
166. de Toranzo EG, Masana M, Castro JA. Administration of benznidazole, a chemotherapeutic agent against Chagas disease, to pregnant rats. Covalent binding of reactive metabolites to fetal and maternal proteins. *Arch Int Pharmacodyn Ther* 1984;**272**: 17–23.
167. Oliveira FC, Chapadeiro E, Alonso MT, Lopes ER, Pereira FE. Chagas disease and pregnancy. I. Incidence of trypanosomiasis and spontaneous abortion in pregnant women with chronic Chagas disease. *Rev Inst Med Trop Sao Paulo* 1966;**8**:184–5.
168. Teruel JR, Nogueira JL. Fetal losses in a high prevalence area of chronic Chagas' disease. *Rev Inst Med Trop Sao Paulo* 1970;**12**:239–44.
169. Castilho EA, Silva GR. Infecçao chagásica materna e prematuridade. *Rev Inst Med Trop Sao Paulo* 1976;**18**:258–60.
170. Hernandez-Matheson IM, Frankowski RF, Held B. Foeto-maternal morbidity in the presence of antibodies to *Trypanosoma cruzi*. *Trans R Soc Trop Med Hyg* 1983;**77**:405–11.
171. Schenone H, Contreras MC, Borgono JM, Rojas A, Villarroel F. Congenital Chagas' disease in Chile. Longitudinal study of the reproductivity of women with or without Chagas' disease and of some parasitological and clinical parameters of them and their corresponding children. *Bol Chil Parasitol* 1985;**40**:24–9.
172. Streiger M, Fabbro D, del Barco M, Beltramino R, Bovero N. Congenital Chagas disease in the city of Santa Fe. Diagnosis and treatment. *Medicina (B Aires)* 1995; **55**:125–32.
173. Zaidenberg M. Congenital Chagas' disease in the province of Salta, Argentina, from 1980 to 1997. *Rev Soc Bras Med Trop* 1999;**32**:689–95.
174. Contreras S, Fernandez MR, Aguero F, Desse DJ, Orduna T, Martino O. Congenital Chagas-Mazza disease in Salta, Argentina. *Rev Soc Bras Med Trop* 1999;**32**:633–6.
175. Blanco SB, Segura EL, Cura EN, Chuit R, Tulian L, Flores I, et al. Congenital transmission of *Trypanosoma cruzi*: an operational outline for detecting and treating infected infants in north-western Argentina. *Trop Med Int Health* 2000;**5**:293–301.
176. Carlier Y, Torrico F. Congenital infection with *Trypanosoma cruzi*: from mechanisms of transmission to strategies for diagnosis and control. *Rev Soc Bras Med Trop* 2003; **6**:767–71.
177. Munoz J, Portus M, Corachan M, Fumado V, Gascon J. Congenital *Trypanosoma cruzi* infection in a non-endemic area. *Trans R Soc Trop Med Hyg* 2007;**101**:1161–2.
178. Klein JO, Baker C, Remington JS, Wilson C. Current concepts of infections of the fetus and newborn infant. In: Remington JS, Klein JO, Wilson C, Baker C, editors. *Infectious diseases of the fetus and newborn infant*. Philadelphia, PA: Elsevier Saunders; 2006. p. 13–25.
179. Munoz M, Thiermann E, Atias A, Acevedo C. Enfermedad de Chagas congenita sintomatica en recien nacidos y lactantes. *Rev Chil Pediatr* 1992;**63**:196–202.
180. Nisida IV, Amato Neto V, Braz LM, Duarte MI, Umezawa ES. A survey of congenital Chagas' disease, carried out at three health institutions in Sao Paulo City, Brazil. *Rev Inst Med Trop Sao Paulo* 1999;**41**:305–11.
181. Oliveira I, Torrico F, Muñoz J, Gascon J. Congenital transmission of Chagas disease: a clinical approach. *Expert Rev Anti Infect Ther* 2010;**8**:945–56.
182. Rubio M, Allende N, Roman C, Ebensperger I, Moreno L. Involvement of the central nervous system in a case of congenital Chagas' disease. *Bol Chil Parasitol* 1967;**22**: 119–22.

183. Saleme AE, Yanicelli GL, Inigo LA, Valperga SM, Alonso E, Paz d E, et al. Congenital Chagas-Mazza disease in Tucuman. Concerning 8 doubtful and 2 probable cases diagnosed in the Dept. of Pediatrics of the S. M. Institute of Maternity during the period October 1967–September 1968. *Arch Argent Pediatr* 1971;**69**:162–9.
184. Vieira GO, Maguire J, Bittencourt AL, Fontes JA. Congenital Chagas' disease. Report of a case with cerebral palsy. *Rev Inst Med Trop Sao Paulo* 1983;**25**:305–9.
185. Howard J, Rubio M. Congenital Chagas' disease. I. Clinical and epidemiological study of 30 cases. *Bol Chil Parasitol* 1968;**23**:107–12.
186. Rubio M. Esophageal involvement in 2 cases of congenital Chagas' disease. *Bol Chil Parasitol* 1968;**23**:157–63.
187. Bittencourt AL, Vieira GO, Tavares HC, Mota E, Maguire J. Esophageal involvement in congenital Chagas' disease. Report of a case with megaesophagus. *Am J Trop Med Hyg* 1984;**33**:30–3.
188. de Almeida MA, Barbosa HS. Congenital Chagas megacolon. Report of a case. *Rev Soc Bras Med Trop* 1986;**19**:167–9.
189. Atias A. A case of congenital chagasic megaesophagus: evolution until death caused by esophageal neoplasm, at 27 years of age. *Rev Med Chil* 1994;**122**:319–22.
190. Costa-Pinto EA, Almeida EA, Figueiredo D, Bucaretchi F, Hessel G. Chagasic megaesophagus and megacolon diagnosed in childhood and probably caused by vertical transmission. *Rev Inst Med Trop Sao Paulo* 2001;**43**:227–30.
191. Atias A, Morales M, Munoz P, Barria M. Ocular involvement in congenital Chagas' disease. *Rev Chil Pediatr* 1985;**56**:137–41.
192. Sologuren Acha R, Oliveira Rezende M, Guzman Heredia R, Coehlo da Silva A, Santos Rezende E, Oliveira Souza C. Prevalence of cardiac arrhythmias during and after pregnancy in women with Chagas disease without apparent heart disease. *Arq Bras Cardiol* 2002;**79**:5–9.
193. Avila WS, Rossi EG, Ramires JA, Grinberg M, Bortolotto MR, Zugaib M, et al. Pregnancy in patients with heart disease: experience with 1,000 cases. *Clin Cardiol* 2003;**26**:135–42.
194. Carlier Y, Pinto Dias JC, Ostermayer Luquetti A, Hontebeyrie M, Torrico F, Truyens C. Trypanosomiase américaine ou maladie de Chagas. In: *Editions Scientifiques et Médicale. Encycl Med Chir, Maladies infectieuses*, Paris: Elsevier SAS; 2002. p. 21.8-505-A-20.
195. Storino R, Auger S, Caravello O, Urrutia MI, Sanmartino M, Jorg M. Chagasic cardiopathy in endemic area versus sporadically infected patients. *Rev Saude Publica* 2002;**36**:755–8.
196. Howard J. Clinical aspects of congenital Chagas disease. In: Pan American Health Organization Scientific Publications. American trypanosomiasis research. 1975. p. 212–15.
197. World Health Organization. Control of Chagas disease. *World Health Organ Tech Rep* 2002;**905**:1–109.
198. Verani JR, Seitz A, Gilman RH, LaFuente C, Galdos-Cardenas G, Kawai V, et al. Geographic variation in the sensitivity of recombinant antigen-based rapid tests for chronic *Trypanosoma cruzi* infection. *Am J Trop Med Hyg* 2009;**80**:410–15.
199. Gamboa-León R, Gonzalez-Ramirez C, Padilla-Raygoza N, Sosa-Estani S, Caamal-Kantun A, Buekens P, et al. Do commercial serologic tests for *Trypanosoma cruzi* infection detect Mexican strains in women and newborns? *J Parasitol* 2011;**97**:338–43.

200. Afonso AM, Ebell MH, Tarleton RL. A systematic review of high quality diagnostic tests for Chagas disease. *PLoS Negl Trop Dis* 2012;**6**:e1881.
201. Guzmán-Gómez D, López-Monteon A, de la Soledad Lagunes-Castro M, Álvarez-Martínez C, Hernández-Lutzon MJ, Dumonteil E, et al. Highly discordant serology against *Trypanosoma cruzi* in central Veracruz, Mexico: role of the antigen used for diagnostic. *Parasit Vectors* 2015;**8**:466.
202. Okumura M, Aparecida dos Santos V, Camargo ME, Schultz R, Zugaib M. Prenatal diagnosis of congenital Chagas' disease (American trypanosomiasis). *Prenat Diagn* 2004;**24**:179−81.
203. Freilij H, Muller L, Gonzalez Cappa SM. Direct micromethod for diagnosis of acute and congenital Chagas' disease. *J Clin Microbiol* 1983;**18**:327−30.
204. Mora M, Sanchez NO, Marco D, Barrio A, Ciaccio M, Segura MA, et al. Early diagnosis of congenital *Trypanosoma cruzi* infection using PCR, hemoculture, and capillary concentration, as compared with delayed serology. *J Parasitol* 2005;**91**:1468−73.
205. Woo PT. The haematocrit centrifuge for the detection of trypanosomes in blood. *Can J Zool* 1969;**47**:921−3.
206. La Fuente C, Saucedo E, Urjel R. The use of microhaematocrit tubes for the rapid diagnosis of Chagas disease and malaria. *Trans R Soc Trop Med Hyg* 1984;**78**:278−9.
207. Moya P, Basso B, Moretti E. Congenital Chagas disease in Cordoba, Argentina: epidemiological, clinical, diagnostic, and therapeutic aspects. Experience of 30 years of follow up. *Rev Soc Bras Med Trop* 2005;**38**(Suppl 2):33−40.
208. de Rissio AM, Scollo K, Cardoni RL. Maternal-fetal transmission of *Trypanosoma cruzi* in Argentina. *Medicina (B Aires)* 2009;**69**:529−35.
209. Russomando G, de Tomassone MM, de Guillen I, Acosta N, Vera N, Almiron M, et al. Treatment of congenital Chagas' disease diagnosed and followed up by the polymerase chain reaction. *Am J Trop Med Hyg* 1998;**59**:487−91.
210. Virreira M, Torrico F, Truyens C, Alonso-Vega C, Solano M, Carlier Y, et al. Comparison of polymerase chain reaction methods for reliable and easy detection of congenital *Trypanosoma cruzi* infection. *Am J Trop Med Hyg* 2003;**68**:574−82.
211. Diez CN, Manattini S, Zanuttini JC, Bottasso O, Marcipar I. The value of molecular studies for the diagnosis of congenital Chagas disease in northeastern Argentina. *Am J Trop Med Hyg* 2008;**78**:624−7.
212. Schijman AG, Bisio M, Orellana L, Sued M, Duffy T, Mejia Jaramillo AM, et al. International study to evaluate PCR methods for detection of *Trypanosoma cruzi* DNA in blood samples from Chagas disease patients. *PLoS Negl Trop Dis* 2011;**5**:e931.
213. Diez C, Manattini S, Imaz MS, Zanuttini JC, Marcipart A. PCR (polymerase chain reaction) in neonatal Chagas disease. An alternative for its early diagnosis? *Medicina (B Aires)* 1998;**58**:436−7.
214. Garcia A, Bahamonde M, Verdugo S, Correa J, Pastene C, Solari A, et al. Infeccion transplacentaria por *Trypanosoma cruzi:* situacion en Chile. *Rev Med Chile* 2001;**129**:330−2.
215. Piron M, Fisa R, Casamitjana N, López-Chejade P, Puig L, Vergés M, et al. Development of a real-time PCR assay for *Trypanosoma cruzi* detection in blood samples. *Acta Trop* 2007;**103**:195−200.
216. Duffy T, Cura CI, Ramirez JC, Abate T, Cayo NM, Parrado R, et al. Analytical performance of a multiplex real-time PCR assay using TaqMan probes for quantification of *Trypanosoma cruzi* satellite DNA in blood samples. *PLoS Negl Trop Dis* 2013;**7**:e2000.

217. Lorca M, Veloso C, Munoz P, Bahamonde MI, Garcia A. Diagnostic value of detecting specific IgA and IgM with recombinant *Trypanosoma cruzi* antigens in congenital Chagas' disease. *Am J Trop Med Hyg* 1995;**52**:512–15.
218. Russomando G, Almiron M, Candia N, Franco L, Sanchez Z, de Guillen I, et al. Implementation and evaluation of a locally sustainable system of prenatal diagnosis to detect cases of congenital Chagas disease in endemic areas of Paraguay. *Rev Soc Bras Med Trop* 2005;**38**(Suppl 2):49–54.
219. Breniere SF, Yaksic N, Telleria J, Bosseno MF, Noireau F, Wincker P, et al. Immune response to *Trypanosoma cruzi* shed acute phase antigen in children from an endemic area for Chagas' disease in Bolivia. *Mem Inst Oswaldo Cruz* 1997;**92**:503–7.
220. Umezawa ES, Nascimento MS, Kesper Jr N, Coura JR, Borges-Pereira J, Junqueira AC, et al. Immunoblot assay using excreted-secreted antigens of *Trypanosoma cruzi* in serodiagnosis of congenital, acute, and chronic Chagas' disease. *J Clin Microbiol* 1996;**34**:2143–7.
221. Freilij HL, Corral RS, Katzin AM, Grinstein S. Antigenuria in infants with acute and congenital Chagas' disease. *J Clin Microbiol* 1987;**25**:133–7.
222. Corral RS, Altcheh J, Alexandre SR, Grinstein S, Freilij, Katzin AM. Detection and characterization of antigens in urine of patients with acute, congenital, and chronic Chagas' disease. *J Clin Microbiol* 1996;**34**:1957–62.
223. Castro-Sesquen YE, Gilman RH, Galdos-Cardenas G, Ferrufino L, Sanchez G, Valencia Ayala E, et al. Use of a novel chagas urine nanoparticle test (chunap) for diagnosis of congenital chagas disease. *PLoS Negl Trop Dis* 2014;**8**:e3211.
224. Azogue E, Darras C. Congenital Chagas in Bolivia: comparative study of the effectiveness and cost of diagnostic methods. *Rev Soc Bras Med Trop* 1995;**28**:3943.
225. Moya P, Moretti E, Paolasso R, Basso B, Blanco S, Sanmartino C, et al. Neonatal Chagas disease: laboratory diagnosis during the first year of life. *Medicina (B Aires)* 1989;**49**:595–9.
226. Russomando G, Sánchez Z, Meza G, de Guillen Y. Shed acute-phase antigen protein in an ELISA system for unequivocal diagnosis of congenital Chagas disease. *Expert Rev Mol Diagn* 2010;**10**:705–7.
227. Mallimaci MC, Sosa-Estani S, Russomando G, Sanchez Z, Sijvarger C, Alvarez IM, et al. Early diagnosis of congenital *Trypanosoma cruzi* infection, using shed acute phase antigen, in Ushuaia, Tierra del Fuego, Argentina. *Am J Trop Med Hyg* 2010;**82**:55–9.
228. Volta BJ, Russomando G, Bustos PL, Scollo K, De Rissio AM, Sánchez Z, et al. Diagnosis of congenital *Trypanosoma cruzi* infection: a serologic test using shed acute phase antigen (SAPA) in mother-child binomial samples. *Acta Trop* 2015;**147**:31–7.
229. de Rissio AM, Riarte AR, García MM, Esteva MI, Quaglino M, Ruiz AM. Congenital *Trypanosoma cruzi* infection. Efficacy of its monitoring in an urban reference health center in a non-endemic area of Argentina. *Am J Trop Med Hyg* 2010;**82**:838–45.
230. Velázquez EB, Rivero R, De Rissio AM, Malagrino N, Esteva MI, Riarte AR, et al. Predictive role of polymerase chain reaction in the early diagnosis of congenital *Trypanosoma cruzi* infection. *Acta Trop* 2014;**137**:195–200.
231. Luquetti AO, Dias J, Prata A. Diagnosis and treatment of congenital infection caused by *Trypanosoma cruzi* in Brazil. *Rev Soc Bras Med Trop* 2005;**38**(Suppl 2):27–8.
232. Altcheh J, Moscatelli G, Mastrantonio G, Moroni S, Giglio N, Marson ME, et al. Population pharmacokinetic study of benznidazole in pediatric Chagas disease suggests efficacy despite lower plasma concentrations than in adults. *PLoS Negl Trop Dis* 2014;**8**:e2907.

233. Moya PR, Paolasso RD, Blanco S, Lapasset M, Sanmartino C, Basso B, et al. Treatment of Chagas' disease with nifurtimox during the first months of life. *Medicina (B Aires)* 1985;**45**:553–8.
234. Altcheh J, Biancardi M, Lapena A, Ballering G, Freilij H. Congenital Chagas disease: experience in the Hospital de Ninos, Ricardo Gutierrez, Buenos Aires, Argentina. *Rev Soc Bras Med Trop* 2005;**38**(Suppl 2):41–5.
235. Chippaux JP, Salas-Clavijo AN, Postigo JR, Schneider D, Santalla JA, Brutus L. Evaluation of compliance to congenital Chagas disease treatment: results of a randomised trial in Bolivia. *Trans R Soc Trop Med Hyg* 2013;**107**:1–7.
236. Suarez E, Alonso-Vega, Torrico F, Cordova M. Integral treatment of congenital Chagas disease: the Bolivian experience. *Rev Soc Bras Med Trop* 2005;**38**(Suppl 2): 21–3.
237. Altcheh J, Moscatelli G, Moroni S, Garcia-Bournissen F, Freilij H. Adverse events after the use of benznidazole in infants and children with Chagas disease. *Pediatrics* 2011;**127**:e212–18.
238. Bianchi F, Cucunubá Z, Guhl F, González NL, Freilij H, Nicholls RS, et al. Follow-up of an asymptomatic Chagas disease population of children after treatment with nifurtimox (Lampit) in a sylvatic endemic transmission area of Colombia. *PLoS Negl Trop Dis* 2015;**9**:e0003465.
239. Castro JA, de Mecca MM, Bartel LC. Toxic side effects of drugs used to treat Chagas' disease (American trypanosomiasis). *Hum Exp Toxicol* 2006;**25**:471–9.
240. Solari A, Ortíz S, Soto A, Arancibia C, Campillay R, Contreras M, et al. Treatment of *Trypanosoma cruzi*-infected children with nifurtimox: a 3 year follow-up by PCR. *J Antimicrob Chemother* 2001;**48**:515–19.
241. Fabbro DL, Danesi E, Olivera V, Codebó MO, Denner S, Heredia C, et al. Trypanocide treatment of women infected with *Trypanosoma cruzi* and its effect on preventing congenital Chagas. *PLoS Negl Trop Dis* 2014;**8**:e3312.
242. Moscatelli G, Moroni S, García-Bournissen F, Ballering G, Bisio M, Freilij H, et al. Prevention of congenital Chagas through treatment of girls and women of childbearing age. *Mem Inst Oswaldo Cruz* 2015;**110**:507–9.
243. Rojo G, Castillo C, Duaso J, Liempi A, Droguett D, Galanti N, et al. Toxic and therapeutic effects of Nifurtimox and Benznidazol on *Trypanosoma cruzi* ex vivo infection of human placental chorionic villi explants. *Acta Trop* 2014;**132**:112–18.
244. Corrêa VR, Barbosa FG, Melo Junior CA, D'Albuquerque e Castro LF, Andrade Junior HF, Nascimento N. Uneventful benznidazole treatment of acute Chagas disease during pregnancy: a case report. *Rev Soc Bras Med Trop* 2014;**47**:397–400.
245. Bisio M, Altcheh J, Lattner J, Moscatelli G, Fink V, Burgos JM, et al. Benznidazole treatment of chagasic encephalitis in pregnant woman with AIDS. *Emerg Infect Dis* 2013;**19**:1490–2.
246. Billot C, Torrico F, Carlier Y. Cost effectiveness study of a control program of congenital Chagas disease in Bolivia. *Rev Soc Bras Med Trop* 2005;**38**(Suppl 2):108–13.
247. Sicuri E, Muñoz J, Pinazo MJ, Posada E, Sanchez J, Alonso PL, et al. Economic evaluation of Chagas disease screening of pregnant Latin American women and of their infants in a non endemic area. *Acta Trop* 2011;**118**:110–17.
248. Torrico F, Alonso Vega C, Billot C, Truyens C, Carlier Y. Relaciones materno-fetales en la infeccion con *T. cruzi* y la implementacion de un programa nacional de deteccion y tratamiento de Chagas congenito en Bolivia. *Enf Emerg* 2007;**9**:9–16.

249. Alonso-Vega C, Billot C, Torrico F. Achievements and challenges upon the implementation of a program for national control of congenital Chagas in Bolivia: results 2004−2009. *PLoS Negl Trop Dis* 2013;**7**:e2304.
250. Soriano-Arandes A, Angheben A, Serre-Delcor N, Treviño-Maruri B, Prat JG, Jackson Y. Control and management of congenital Chagas disease in Europe and other non-endemic countries: current policies and practices. *Trop Med Int Health* 2016;**21**:590−6.
251. Garcia-Bournissen F, Altcheh J, Panchaud A, Ito S. Is use of nifurtimox for the treatment of Chagas disease compatible with breast feeding? A population pharmacokinetics analysis. *Arch Dis Child* 2010;**95**:224−8.
252. García-Bournissen F, Moroni S, Marson ME, Moscatelli G, Mastrantonio G, Bisio M, et al. Limited infant exposure to benznidazole through breast milk during maternal treatment for Chagas disease. *Arch Dis Child* 2015;**100**:90−4.
253. Vela-Bahena LE, Vergara R, Vite L, Ramos C. Postpartum treatment without interrupting breastfeeding in a patient with Chagas disease. *Ginecol Obstet Mex* 2015;**83**:487−93.
254. Forés R, Sanjuán I, Portero F, Ruiz E, Regidor C, López-Vélez R, et al. Chagas disease in a recipient of cord blood transplantation. *Bone Marrow Transplant* 2007;**39**:127−8.
