# Supplementary material for: Chagas disease screening in pregnant Latin American women: Adherence to a systematic screening protocol in a non-endemic country
Source: PLoS Negl Trop Dis. 2021 Mar 24;15(3):e0009281. doi: 10.1371/journal.pntd.0009281 (PMC8021187; doi:10.1371/journal.pntd.0009281)
Supplement: Attachment — Submitted filename: PNTD-D-20-01929.docx [file pntd.0009281.s001.docx]

Llenas-Garcia et al. report adherence to screening for Chagas disease in Latin-American pregnant women in 10 health departments in Alicante province (Spain) between 2014-2018.

The study was retrospective using a cross-check between number of deliveries among LA pregnant women and serology made in the same period.

The results are interesting but there are several flawness:

1) In four departments data were available only for the period 2016-18 (San Juan, Marina Baixa, Elche General) or 2015-18 (Torrevieja) and not for the entire period of study.
2) Methods of screening were made by using different methods: single CLIA in five departments (Orihuela, Elx-Crevillente, Alicante general, San Juan, Torrevieja); one of two ICT in two depts (Alcoy, Denia); two methods (CLIA+ICT) in three depts (Marina Baixa, Elche general, Elda). Authors state that a second technique was performed if the first sample was positive. However, it is not reported what method was used nor how many samples wer not confirmed or how many indeterminate results wer observed.
3) To estimate the number of missing cases they used data gathered from two studies (Moncayo Mem Inst Oswaldo Cruz 2009; Requena-Mendez Plos Negl Trop Dis 2015); I don’t think is correct (table 3 seroprevalence for Argentina, Bolivia, Brazil, Chile, Colombia, Ecuador, El Salvador, Honduras, Mexico, Nicaragua,Uruguay, Paraguay, Venezuela from Requena-Mendez; Costa Rica, Panama, Perù from Moncaya; seroprevalence for Guatemala,?).
4) To estimate the number of infected newborns the rate of the study by Murcia (13.8%) seems too high (in a recent meta-analysis conducted in non-endemic countries was 3.5%: Colombo V et al. J Travel Med 2020 Sept 18;taaa170).

Other remarks:

**Title**: add the word “adherence” before “systematic screening protocol”

**Introduction**: page 5 line 84: oral transmission is not cited; page 5 line 87: two licensed drugs (is not correct the term licensed; in many countries one or both drugs are not licensed)

Line 102 cite the meta-analysis conducted in non-endemic countries (Colombo V et al. J Travel med 2020). Page 6 line 110: reference 13 is about Bolivia (the study refers to area without vectorial transmission but in an endemic country).

**Study design and data collection**: page 7 line 143: it is unclear why serologies are considered from May 2013

**Figure 1**: as it stands is unuseful; I suggest to redarwn putting a smaller map of Spain and a blow-up of the province of Alicante indicating the location of the 10 hospitals.

**Table 1**: add a column reporting the different methods used (CLIA, CLIA+ICT, ICT); total prevalence are not correct: 30 (1,8%) and 24 (10,4%) should be : 30 (2,3%) and 24 (18,9%).

**Discussion**: It is too long and several statements are not supported by the study’s results (page 13 line 246-254). The high rate of follow-up loss should be commented more deeply (it is a problem raised in many studies performed in Europe; for instance Repetto EC et al. Plos Negl Trop Dis 2015). You should focus more on your results and try to explain why no improvement was observed with previous studies in the same area (references 18 and 19).

Please comment on the different methods used to diagnose Chagas disease and their sensitivity/specificity.
