# Supplementary material for: Chagas disease screening in pregnant Latin American women: Adherence to a systematic screening protocol in a non-endemic country
Source: PLoS Negl Trop Dis. 2021 Mar 24;15(3):e0009281. doi: 10.1371/journal.pntd.0009281 (PMC8021187; doi:10.1371/journal.pntd.0009281)
Supplement: Attachment — Submitted filename: Letter to editors with answers to reviewers_final_2.docx [file pntd.0009281.s002.docx]

Jara Llenas-García, PhD

Hospital Vega Baja-FISABIO
Crta Orihuela-Almoradí s/n
03340 Orihuela (Alicante) Spain

Telephone number: +34652584257

Email: jarallenas@gmail.com

Erica Silberstein

Guest Editor

PLOS Neglected Tropical Diseases

Alain Debrabant

Deputy Editor

PLOS Neglected Tropical Diseases

26th January 2020

**To the editors of PLOS Neglected Tropical Diseases**

**Response-to-reviewers letter and revised manuscript entitled “Chagas disease screening in pregnant Latin American women: systematic screening protocol in a non-endemic country" Ref: PNTD-D-20-01929**

Dear Editors,

This document provides point-by-point responses to the reviewers’ comments. Their comments, reproduced in *italics*, are followed by our responses and, finally by the additional text and changes (**in bold blue**) to the manuscript first submitted. Please note that line numbers in this letter are referred to the tracked changes version. We thank the three reviewers for their comments which have much helped improve our paper.

**Reviewer #1:** *The MS PNTD-D-20-01929 evaluates the implementation of the CD screening program of Latin American pregnant women in the Spanish province of Alicante between 2014 and 2018.Though such program has been improved since the previous evaluation of 2009-2010, the authors analyze the reasons of some persisting failures in order to improve it still more. Such analyze has an evident public health interest.

Our main comments concern:*

*1) The comparison with the previous evaluation of 2009-2010 (just mentioned l. 235), would be advantageously emphasized and discussed;*

- We thank the reviewer for this comment. We have added a sentence in the discussion, line 290:

**“The absence of improvement in part might be due to the lack of an institutional program surveillance, with no feedback given to the program implementers**. **On the contrary, the systematic surveillance program** in the region of Catalonia concluded that the congenital CD screening program was successful, with an overall coverage rate of 83.5% in 2010-15 and substantial improvements from 2010 (68.4%) to 2015 (88.6%)[25]….”

*2) Since 42% of seropositive mothers were treated before their pregnancy, it would be interesting to specify the conditions of such treatment (used drug, doses, timing…) and, if possible, their serological post-treatment evolution. This is all the more important that treatment before pregnancy is recommended to avoid congenital transmission, and that no congenital infection was detected  among such mothers;*

- We have reviewed those conditions and have added a sentence in the Results section, line 250:

**“Treatment had been done a mean of 3.2 (SD 2.1) years before delivery, in most of them (8/9) with benznidazole 5mg/kg/day for 60 days; only one had negative serologies in the post-treatment follow-up.”**

*3) Table 1 indicates a total of 1.8% of positive serology, whereas the data are 30/1323, i.e. 2.3%; to be corrected;*

- We thank the reviewer for this accurate comment. We have corrected it also according to the next comment (see modifications after comment number 4).

*4) The timing of sampling for serology in pregnant women should be specified.*

- We are in debt with the reviewer for this comment. We had not analysed timing of sampling for serology. Once we have done the analysis we have realized that 10.9% of serologies were collected ≥10 days post-partum and therefore could not be considered a perinatal screening. We have therefore corrected the material and methods section, the numbers in the results section and in the abstract and summary. We have also added a comment on serology timing in the results section, line 230:

**“Of the 1178 serologies, 589 (50.0%) were done in the first trimester of pregnancy, 220 (18.7%) in the second trimester and 369 (31.3%) in the third trimester or first 10 days postpartum.”**

Corrections in the abstract (line 51):

**“Results:** There were **3026** deliveries in Latin American women during the study period; **1178 (38.9%)** underwent CD serology. Screening adherence ranged from **17.2% to 59.3%** in the different health departments and was higher in Bolivian women **(48.3%).** **Twenty-six deliveries (2.2%)** had a positive screening; CD was confirmed in **23 (2%)** deliveries of **21** women. Bolivians had the highest seroprevalence **(21/112; 18.7%),** followed **by Colombians (1/333; 0.3%)** and Ecuadorians **(1/348; 0.3%).** Of **21** CD-positive women (**19** Bolivians, **1** Colombian, **1** Ecuadorian), infection was already known in **12 (57.1%),** and **9 (42.9%)** had already been treated. Only **1 of the 12** untreated women **(8.3%)** was treated postpartum. Follow-up started in **20 of the 23 (87.0%)** neonates but was completed only in **11 (47.8%);** no cases of congenital transmission were detected. Among the **1848** unscreened deliveries, we estimate **43** undiagnosed cases of CD and **1 to 2** undetected cases of congenital transmission**.”**

Corrections in Authors’summary (line 72):

“In our study we analyzed adherence to that recommendation in Alicante province from 2014 to 2018, finding that it is quite low (**38.9%** overall, **48.3%** in Bolivians) and heterogeneous between health departments. Among unscreened pregnant women during the study period, we estimate that there could be **43** undiagnosed cases of CD and **1 to 2** undetected infections in infants. We also observed very low adherence to treatment after delivery in CD-diagnosed, untreated women (**8.3%),** and a low rate of completed follow-up in newborns at risk of vertical infection **(47.8%).**

Sentence added in methods, line 159:

**“We included serologies sampled before delivery and until 10 days post-partum.”**

Changes in Results section are numerous, as we are now including 1178 serologies instead of 1323. We are not highlighting every change in this letter but they can be easily followed at the revised version with tracked changes. We include here main changes in table 1

**“Table 1.** Adherence to the systematic screening protocol of Latin American pregnant women by hospital, 2014 to 2018

|  | | |  | | **Total sample** | | |  | **Bolivian women** | | |
| --- | --- | --- | --- | --- | --- | --- | --- | --- | --- | --- | --- |
| **Hospital** | **N deliveries** | | | **Serological techniques** | | **N (%) serologies** | **N CD serologies-positive** |  | **N deliveries** | **N (%) serologies** | **N CD serologies-positive** |
| Alicante- General | | 783 | | CLIA→ELISA | | **464 (59.3)** | **9** |  | 52 | 34(65.4) | 6 |
| San Juan | | **203** | | CLIA→ELISA | | **35 (17.2)** | **0** |  | 13 | 4 30.8) | **0** |
| Marina Baixa | | 269 | | CLIA+ICT | | **129 (47.9)** | **9** |  | 25 | 16 (64.0) | **9** |
| Orihuela | | 255 | | CLIA→ICT | | **73 (28.6)** | 3 |  | 48 | 19 (39.6) | 3 |
| Denia | | 262 | | ICT→CLIA | | **87 (33.2)** | 2 |  | 37 | 16 (43.2) | 1 |
| Torrevieja | | 367 | | CLIA→ICT | | **70 (19.1)** | 1 |  | 7 | 3 (42.9) | 0 |
| Elda | | 261 | | CLIA+ICT | | **80 (30.6)** | 0 |  | 15 | 5 (33.3) | 0 |
| Elche- General | | 176 | | CLIA+ICT | | **69 (39.2)** | 1 |  | 7 | 2 (28.6) | 1 |
| Elche-Crevillente | | 330 | | CLIA→ICT | | **141 (42.7)** | **1** |  | 16 | 11 (68.7) | **1** |
| Alcoy | | 120 | | ICT+ICT | | **30 (25.0)** | **0** |  | 12 | 2 (16.7) | **0** |
| ***TOTAL*** | | ***3026*** | |  | | ***1178 (38.9)*** | ***26 (2.2%)*** |  | ***232*** | ***112 (48.3)*** | ***21 (18.7%)*** |

Changes in the Discussion, are:

Line 269: However, we observed adherence to this systematic screening protocol of just **38.9%** in Latin American pregnant women from 2014 to 2018.

Line 288: In Alicante-General, the coverage rate improved from 41.2% in 2008-2012 to **59.3%** in 2014-2018, while in Elche-General the rate was steady (40% vs **39.2%)** between 2008-2011 and 2014-2018

*Minor comments concern:*

*1) The term “vertical” transmission (including breast milk transmission) should be replaced by “congenital” or “conatal” (limited to trans-placental transmission) (keywords, l. 42, 65…);*

- We thank the reviewer for this appreciation. We have replaced the word vertical by congenital where appropriate (keywords, lines 44, 61, 62, 67, 70, 256, 260, 262 and 402)

*2) References should be added, such as:  PLoS NTD. 2019, 13(10), e0007694 (l. 92, 98, 107, 110, 114..); Brit J Gyn Obst 2014, 121(1), 22-33 (l. 102…); Chap 23, p517-559 in American trypanosomiasis-Chagas disease. One hundred years of research. Elsevier, 2017 ,2 edition, ISBN 978-0-12-801029-7 (l. 306); Cur Trop Med Rep 2020, 7 (4), 172-182 (l. 306)…;*

- We thank the reviewer for the recommendation. We have added reference by Carlier et al [10] in lines 104 and 119.

“To control transmission, it is essential to test all pregnant women living in or migrating from endemic countries[9,**10**]”

“Serological screening in pregnant women by rapid diagnostic tests and examination of babies born from seropositive mothers is a suitable strategy to detect and prevent congenital Chagas disease in non-endemic areas **of both endemic and non-endemic countries**[**10,**15–17]”

- The meta-analysis by Howards et al [14] has been added in line 111 and a new sentence has been added:

**“A previous meta-analysis reported a higher transmission rate in endemic countries (5%) than in non-endemic ones (2.7%)[14].”**

- Reference by Carlier, Schijman & Kemmerling [48] has been added in line 323:

“There is also a need to standardize, expand and reinforce CD screening in all women of Latin American origin who are of childbearing age (pregnant or not); treating childbearing age women is a useful strategy to decrease vertical transmission in potential future pregnancies, since there is a correlation between the parasitemia of the mother and congenital infection[47,**48**].”

- Reference *Chap 23, p517-559 in American trypanosomiasis-Chagas disease. One hundred years of research. Elsevier, 2017 ,2 edition, ISBN 978-0-12-801029-7 (l. 306)* has not been added because unfortunately we could not read the proposed chapter.

*3) Table 3 should specify the source of estimated prevalences in addition to their mention in Material and Methods.*

- References have been added to table 3 that now is:

**Table 3.** Estimated cases of undiagnosed CD cases among unscreened pregnant women, 2014 to 2018

| **Nationality** | **Unscreened deliveries** | **Estimated seroprevalence (%)[8,24]** | **Estimated n missed cases in pregnant women** |
| --- | --- | --- | --- |
|  |  |  |  |
|  |  |  |  |

**Reviewer #2:**

Line 82: `Vector-borne transmission is the main route of contagion in endemic areas”

- We have changed the sentence (line 87) as suggested:

“Vector-borne transmission is the main route of contagion **in endemic areas**, although infection can also spread via blood transfusion, organ transplantation, **orally** or from mother to child[1].”

Line 87: Please add women in childbearing age in treatment indications

- We have added the suggestion in line 93:

“Antiparasitic treatment with one of the two **approved** drugs (benznidazole and nifurtimox) is indicated for acute and congenital CD, reactivated infections, **women of childbearing age** and chronic disease in children.”

Line 111: PCR of blood from umbilical cord is not a validated method for screening yet and false positive results have been reported with this technique. (Basile Luca, Ciruela Pilar, Requena-Mendez Ana, Euro Surveill. 2019;24(26):pii=1900011. <https://doi.org/10.2807/1560-7917.ES.2019.24.26.19-00011>) and (Buekens, P., Am. J. Trop. Med. Hyg., 98(2), 2018, pp. 478–485 doi:10.4269/ajtmh.17-0516) About infants not diagnosed at birth, you should mention the availability of parasitic direct visualization methods for diagnosis, in infants younger than 9 months of age. (Carlier et al 2019 Plos Neglect Trop Dis <https://doi.org/10.1371/journal.pntd.0007694>)

- We thank the reviewer for the accurate comment. We have made the following changes, line 119:

“In Spain, experts recommend prenatal serological screening followed by microscopic examination or polymerase chain reaction (PCR) of cord blood from infants of seropositive mothers at birth **and by one month of age**. **Molecular tests are not well validated for congenital transmission diagnosis and can have false positives, especially in blood samples collected from umbilical cord. However, they can be considered as uptaking tests in order to prevent patient lost-to-follow-up, when (as in our region) parasitological techniques are not available or reliable[10].** For infants not diagnosed at birth, **direct parasitological methods are useful for diagnosis in infants <9 months of age**, while conventional serology is recommended at 9 months of age[15].”

Line 151: It is not clear if serologic results are confirmed by two different techniques –or a third if indeterminated results. Please clarify this.

- It has been clarified in line 172:

“**In those using only one serological technique, a second technique was performed if the first one was positive (immunochromatography in Orihuela, Elx-Crevillente and Torrevieja, ELISA in Alicante-General, and San Juan, and CLIA in Denia). Discordant results were solved using with a third technique, an indirect immunofluorescence assay done in a National Reference Laboratory**. CD diagnosis was confirmed by positive results from two different serological techniques using different antigens. **Indeterminate results in the first serology were considered as negative**.”

Line 155: A second technique was only performed if first was positive? Please consider that diagnosis is discharged with two different techniques being negative. (Carlier et al 2019 Plos Neglect Trop Dis <https://doi.org/10.1371/journal.pntd.0007694>)

- Unfortunately, we do not fully agree with this comment. In the recommendations by Carlier et al is stated that: “The highly sensitive chemiluminescent immunoassay (CMIA) [[19](https://journals.plos.org/plosntds/article?id=10.1371/journal.pntd.0007694#pntd.0007694.ref019)] can be used for screening as a stand-alone test”. In our study most facilities performing only one technique (5/6) used highly sensitive CMIA, that has shown a 100% sensitivity and 96.6% specificity. We added a comment on tests’ sensitivity in the discussion (line 316):

“**In our region several serological diagnostic tests are used. CLIA Architect sensitivity is reported to be 100% and specificity 96.6% and has been proposed as a single test for diagnosis[10,36].”**

Line 161: Please justify the rational for serology conducted at 1 month, as passage of mother antibodies to newborn’s blood could cause a positive serology result in not infected patients at this time. Also, serology after 9 months of age should be performed regardless previous negative or positive results. I suggest a flow chart explaining the proposed follow-up.

- We thank the reviewer for this appreciation. There was a mistake, as serology is not done at 1 month but a repeated PCR. We changed the sentence (line 183) to:
- “Follow-up of the exposed newborn includes PCR **± parasitological methods at birth and at 1 month, and serology and PCR** at 9-12 months[15,18].”

Line 189: Adherence to screening was not previously defined. I suggest including this definition.

- We changed the term adherence to screening to percentage of pregnancies screened (line 215).

“**Percentage of pregnancies screened** ranged from **59.3% (95% CI 55.8%-62.7%)** in Alicante-General to **17.2% (95% CI 12.0%-22.4%) in San Juan**, and it was higher in Bolivian women (**48.3%; 95% CI 41.7%-54.9%; table 1).**

Line 219: Please detail “workup for CD”

- A definition of CD workup has been added in Methods, line 185:

**“CD workup of the positive mothers includes at least a complete medical history, physical examination, T.cruzi PCR, echocardiogram, electrocardiogram and chest X-ray.”**

Line 221: Please explain age of this “clinical visit” and which test was performed (PCR? Serology?) with technique description.

- We have explained age of that visit although it was not homogeneous (nor the procedures) between centres. We have added a sentence explaining what it consisted of in most centers (line 254).

“T**wenty** of the **23** newborns (**87.0%**) had at least one clinical visit to assess CD transmission (**most of them at 1 month of age to check for at birth PCR results**), but only **11 (47.8%)** completed all clinical visits.”

Table 3. Line 228: I suggest explaining that estimated seroprevalence for each country varies widely through different regions within each country.

- We fully agree with the reviewer and have added it as a limitation of the study (line 394):

**“Country seroprevalence used to estimate missing cases could not be accurate as seroprevalence varies widely through different regions within each country”.**

Line 246: Please state that serology could be made at any trimester of pregnancy in non-endemic regions

- We have added the suggested statement (line 274), although we feel is somewhat unnecessary and we wrote first-trimester because is as it is in the regional protocol.
- “Our low adherence rate may be attributable to several factors. First, screening requires midwives or obstetricians to remember to add CD testing to first-trimester serologies in pregnant women from endemic countries (**although serology could be made at any trimester of pregnancy in non-endemic regions**).”

Line 273: According to NIH recommendations, the studies performed on breast milk with these drugs have sufficient number of patients.

- We have changed that part of the discussion (line 327):

“Moreover, most guidelines recommend not treating lactating women due to concerns about benznidazole or nifurtimox transfer into breast milk[15], **although some studies have found very limited transference of benznidazole or nifurtimox into breast milk[40,41].**”

Line 283: Diagnose should be guided by WHO recommendations. Take note that PAHO/WHO consider PCR as an experimental method yet to be validated (Carlier et al 2019 Plos Neglect Trop Dis <https://doi.org/10.1371/journal.pntd.0007694>).

- We have shortened the discussion as suggested by other reviewer and the sentence has been deleted. However, we added a sentence in the introduction (line 122):

**“Molecular tests are not well validated for congenital transmission diagnosis and can have false positives, especially in blood samples collected from umbilical cord. However, they can be considered as uptaking tests in order to prevent patient lost-to-follow-up, when (as in our region) parasitological techniques are not available or reliable[10].”**

Line 286: There is evidence of false positive PCR results in neonates and this technique is still not well validated. (Basile Luca, Ciruela Pilar, Requena-Mendez Ana, Euro Surveill. 2019;24(26):pii=1900011. <https://doi.org/10.2807/1560-7917.ES.2019.24.26.19-00011>) (Buekens, P., Am. J. Trop. Med. Hyg., 98(2), 2018, pp. 478–485 doi:10.4269/ajtmh.17-0516)

- We thank the reviewer for this appreciation. Please check previous answer.

**Reviewer #3:**

*Llenas-Garcia et al. report adherence to screening for Chagas disease in Latin-American pregnant women in 10 health departments in Alicante province (Spain) between 2014-2018.*

*The study was retrospective using a cross-check between number of deliveries among LA pregnant women and serology made in the same period.*

*The results are interesting but there are several flawness:*

1) *In four departments data were available only for the period 2016-18 (San Juan, Marina Baixa, Elche General) or 2015-18 (Torrevieja) and not for the entire period of study.*

- We agree with the reviewer that this is a flawness of our study but, unfortunately, we have no previous data available as those hospitals did not have electronic microbiological registries until 2015-2016. We have added a comment on the study limitations (line 392):

**“Also, we only have data for the whole study period of six hospitals, while in the other four only data from the last years were collected, as previous electronic microbiological reports were not recorded”**.

2) *Methods of screening were made by using different methods: single CLIA in five departments (Orihuela, Elx-Crevillente, Alicante general, San Juan, Torrevieja); one of two ICT in two depts (Alcoy, Denia); two methods (CLIA+ICT) in three depts (Marina Baixa, Elche general, Elda). Authors state that a second technique was performed if the first sample was positive. However, it is not reported what method was used nor how many samples wer not confirmed or how many indeterminate results wer observed.*

- We have added information on the second technique used and the protocol in case of serodiscordant results (line 172).

**“In those using only one serological technique, a second technique was performed if the first one was positive (immunochromatography in Orihuela, Elx-Crevillente, and Torrevieja, ELISA in Alicante-General and San Juan and CLIA in Denia). Discordant results were solved using with a third technique, an indirect immunofluorescence assay done in a National Reference Laboratory.”**

- Number of samples confirmed is reported in the results section, line 238:

“CD was confirmed with a second serological technique in 23 deliveries (2%, 21 women).)”.

- The number of indeterminate results has been added (line 234), as an explication in methods (line 178):

“CD diagnosis was confirmed by positive results from two different serological techniques using different antigens. **Indeterminate results in the first serology were considered as negative. “**

“**There were 3 deliveries with indeterminate results in the first serology.”**

3) *To estimate the number of missing cases they used data gathered from two studies (Moncayo Mem Inst Oswaldo Cruz 2009; Requena-Mendez Plos Negl Trop Dis 2015); I don’t think is correct (table 3 seroprevalence for Argentina, Bolivia, Brazil, Chile, Colombia, Ecuador, El Salvador, Honduras, Mexico, Nicaragua,Uruguay, Paraguay, Venezuela from Requena-Mendez; Costa Rica, Panama, Perù from Moncaya; seroprevalence for Guatemala,?).*

- We thank the reviewer for this accurate observation. Guatemala estimated seroprevalence as for Moncayo et al is 0.84% and therefore the estimated number of missing cases in pregnant women is 0.03. The table has been corrected as follows:

**“Table 3.** Estimated cases of undiagnosed CD cases among unscreened pregnant women, 2014 to 2018

| **Nationality** | **Unscreened deliveries** | **Estimated seroprevalence (%)** | **Estimated n missed cases in pregnant women** |
| --- | --- | --- | --- |
| Guatemala | 3 | **0.84** | **0.03** |
| ***TOTAL*** | ***1848*** | ***—*** | ***42.79*** |

4) *To estimate the number of infected newborns the rate of the study by Murcia (13.8%) seems too high (in a recent meta-analysis conducted in non-endemic countries was 3.5%: Colombo V et al. J Travel Med 2020 Sept 18;taaa170).*

- We thank the reviewer for this comment. The meta-analysis by Colombo et al was not yet published when we drafted the article. We have added its information and used it for our estimation on unnoticed vertical transmission. In methods (line 189) now it says:

“To estimate the number of infected newborns, we used vertical transmission rates **reported in a recent meta-analysis[13]. “**

And in results we have changed the sentence accordingly (line 260):

“Assuming a vertical transmission rate of **3.8% (95%CI: 2.4-5.1%)[13],** there would be **1.6 (95%CI: 1.0-2.2)** estimated cases of undetected **congenital** transmission. “

We have also changed the Abstract (line 61):

“Among the **1848** unscreened deliveries, we estimate **43** undiagnosed cases of CD and 1 to **2** undetected cases of **congenital** transmission.”

And the Author’s summary (line 74):

“Among unscreened pregnant women during the study period, we estimate that there could be **43** undiagnosed cases of CD and 1 to **2** undetected infections in infants.”

*Other remarks:*

*Title: add the word “adherence” before “systematic screening protocol”*

- We have added adherence to a before systematic screening protocol so the title (line 2) now reads:

“Chagas disease screening in pregnant Latin American women: **adherence to a** systematic screening protocol in a non-endemic country”

*Introduction: page 5 line 84: oral transmission is not cited; page 5 line 87: two licensed drugs (is not correct the term licensed; in many countries one or both drugs are not licensed)*

- We have added oral transmission to the sentence (line 89):

“Vector-borne transmission is the main route of contagion **in endemic areas**, although infection can also spread via blood transfusion, organ transplantation, **orally** or from mother to child[1].”

- The term licensed has been changed for approved (line 92). The sentence now reads: “Antiparasitic treatment with one of the two **approved** drugs (benznidazole and nifurtimox) is indicated for acute and congenital CD, reactivated infections, **women of childbearing age** and chronic disease in children.”

*Line 102 cite the meta-analysis conducted in non-endemic countries (Colombo V et al. J Travel med 2020).*

- We have cited the meta-analysis and changed the sentence (line 107) to:

“**Congenital T. cruzi infection rate ranges in different studies from 2%[11] to 13.8%[5]; a recent meta-analysis reported vertical transmission rate of 3.8% (CI95%: 2.4-5.1%) when poor quality studies were excluded[13].”**

*Page 6 line 110: reference 13 is about Bolivia (the study refers to area without vectorial transmission but in an endemic country).*

- We thought that “non-endemic areas” was correct enough but we have added of both endemic and non-endemic countries for clarification (line 119). The sentence now says:

“Serological screening in pregnant women by rapid diagnostic tests and examination of babies born from seropositive mothers is a suitable strategy to detect and prevent congenital Chagas disease in non-endemic areas **of both endemic and non-endemic countries**[**10, 15–17]”**

*Study design and data collection: page 7 line 143: it is unclear why serologies are considered from May 2013*

- A clarification sentence (line 158) has been added:

“**We searched for serologies from the 8 months prior to the first delivery record to include all those done at the prenatal consultation.”**

*Figure 1: as it stands is unuseful; I suggest to redarwn putting a smaller map of Spain and a blow-up of the province of Alicante indicating the location of the 10 hospitals.*

- We thank the reviewer for this suggestion. Figure 1 has been modified accordingly.

*Table 1: add a column reporting the different methods used (CLIA, CLIA+ICT, ICT); total prevalence are not correct: 30 (1,8%) and 24 (10,4%) should be : 30 (2,3%) and 24 (18,9%).*

- We thank the reviewer for this accurate comment. Total prevalence has been corrected in the table and a column with the serological techniques used in each center has been added. The table now shows:

**Table 1.** Adherence to the systematic screening protocol of Latin American pregnant women by hospital, 2014 to 2018

|  | |  | | **Total sample** | | |  | **Bolivian women** | | |
| --- | --- | --- | --- | --- | --- | --- | --- | --- | --- | --- |
| **Hospital** | **N deliveries** | | **Serological techniques** | | **N (%) serologies** | **N CD serologies-positive** |  | **N deliveries** | **N (%) serologies** | **N CD serologies-positive** |
| Alicante- General | 783 | | **CLIA→ELISA** | | 464 (59.3) | 9 |  | 52 | 34(65.4) | 6 |
| San Juan | 203 | | **CLIA→ELISA** | | 35 (17.2) | 0 |  | 13 | 4 30.8) | 0 |
| Marina Baixa | 269 | | **CLIA+ICT** | | 129 (47.9) | 9 |  | 25 | 16 (64.0) | 9 |
| Orihuela | 255 | | **CLIA→ICT** | | 73 (28.6) | 3 |  | 48 | 19 (39.6) | 3 |
| Denia | 262 | | **ICT→CLIA** | | 87 (33.2) | 2 |  | 37 | 16 (43.2) | 1 |
| Torrevieja | 367 | | **CLIA→ICT** | | 70 (19.1) | 1 |  | 7 | 3 (42.9) | 0 |
| Elda | 261 | | **CLIA+ICT** | | 80 (30.6) | 0 |  | 15 | 5 (33.3) | 0 |
| Elche- General | 176 | | **CLIA+ICT** | | 69 (39.2) | 1 |  | 7 | 2 (28.6) | 1 |
| Elche-Crevillente | 330 | | **CLIA→ICT** | | 141 (42.7) | 1 |  | 16 | 11 (68.7) | 1 |
| Alcoy | 120 | | **ICT+ICT** | | 30 (25.0) | 0 |  | 12 | 2 (16.7) | 0 |
| ***TOTAL*** | ***3026*** | |  | | ***1178 (38.9)*** | ***26 (2.2%)*** |  | ***232*** | ***112 (48.3)*** | ***21 (18.7%)*** |

**CD: Chagas disease; CLIA: chemiluminescence immunoassay; ICT: immunochromatography; ELISA: enzyme-linked immunosorbent assay**

*Discussion: It is too long and several statements are not supported by the study’s results (page 13 line 246-254). The high rate of follow-up loss should be commented more deeply (it is a problem raised in many studies performed in Europe; for instance Repetto EC et al. Plos Negl Trop Dis 2015). You should focus more on your results and try to explain why no improvement was observed with previous studies in the same area (references 18 and 19).*

- We thank the reviewer for this comment. We have shortened the discussion, we have added a comment on the high rates of lost-to-follow-up, we have included the suggested citation and we have tried to explain the reason for no improvement. Main changes in the discussion are:

Line 290_ “**The absence of improvement in part might be due to the lack of an institutional program surveillance, with no feedback given to the program implementers**. **On the contrary, the systematic surveillance program** in the region of Catalonia concluded that the congenital CD screening program was successful, with an overall coverage rate of 83.5% in 2010-15 and substantial improvements from 2010 (68.4%) to 2015 (88.6%)[25]….

Line 278**…Workshops with midwives and automatic alerts** for CD screening based on nationality in the electronic health record could improve compliance with the protocol. **Including a serology at delivery in cases not previously screened could help improve screening rates…..**

Line 311….In our study, T. cruzi seroprevalence was somewhat lower than in previous studies **where ranged from 1.5% to 12% [16,25,28–33**], probably due to the low percentage of Bolivian women included. In the largest study from Catalonia, the overall prevalence was 2.8 cases per 100 pregnancies per year but reached 15.8 in Bolivian women[25]….

Line 325… **High rates of lost-to-follow-up have been reported in other European programs [34] and are a threat to CD control in Europe….**

Line 365. **Affected communities’ involvement is key to reduce lost-of-follow-up rates as is the creation of a comprehensive community-based CD program that includes a systematic surveillance system**

*Please comment on the different methods used to diagnose Chagas disease and their sensitivity/specificity.*

- A sentence has been added in the discussion (line 316):
- **“In our region several serological diagnostic tests are used. CLIA Architect sensitivity is reported to be 100% and specificity 96.6% and has been proposed as a single test for diagnosis[10,36]. ICT sensitivity range from 92% to 98%, while specificity is reported to be between 97% and 100%[37]. A recent meta-analysis yielded a pooled sensitivity and specificity of ICT under field conditions of 96.6% (95% CI 91.3–98.7%) and 99.3% (95% CI 98.4–99.7%), respectively[38].”** **ELISA and IFI have shown a sensitivity of 97-100%[37].**

Finally, as a result of this review process we have included a new author, Maria Montserrat Ruíz-García, that has greatly contributed with her laboratory data and to discuss microbiological techniques’ performance. We have also corrected and error in the affiliation of author Guillermo Telenti. Now, the list of authors and affiliations is:

# Authors and Affiliations:

Jara Llenas-García^1,2^, Philip Wikman-Jorgensen^3^, Concepción Gil-Anguita^4^, Violeta Ramos- Sesma^5^, Diego Torrús-Tendero^6,7^, Raquel Martínez-Goñi^8^, Mónica Romero-Nieto^2,9^, Javier García-Abellán^10^, Mª José Esteban-Giner^11^, Karenina Antelo^12^, María Navarro-Cots^13^, Fernando Buñuel^14^, Concepción Amador^4^, Josefa García-García^15^, Isabel Gascón^16^, Guillermo Telenti**^10^**, Encarna Fuentes-Campos^17^, Ignacio Torres^18^, Adelina Gimeno-Gascón^19^, **María Montserrat Ruíz-García^20^**, Miriam Navarro^21^, José-Manuel Ramos-Rincón^2,6^.

1. Internal Medicine Department. Hospital Vega Baja de Orihuela (Alicante, Spain)-Foundation for the Promotion of Health and Biomedical Research of the Valencia Region (FISABIO).
2. Clinical Medicine Department. University Miguel Hernández de Elche, Alicante, Spain
3. Internal Medicine Department. University Hospital San Juan de Alicante (Alicante, Spain)-FISABIO.
4. Internal Medicine Department. Hospital Marina Baixa de Villajoyosa (Alicante, Spain).
5. Internal Medicine Service, HLA Inmaculada Hospital, Granada (Spain)
6. Reference Unit of Imported Diseases and International Health. Alicante General University Hospital (Alicante, Spain). Biomedical and Health Research Institute of Alicante (ISABIAL)
7. Parasitology Area. University Miguel Hernández de Elche, Alicante, Spain
8. Internal Medicine Department. University Hospital Vinalopó de Elche (Alicante, Spain).
9. Internal Medicine Department. Elda General University Hospital (Alicante, Spain)-FISABIO.
10. Infectious Diseases Unit. Elche General University Hospital-FISABIO.
11. Internal Medicine Department. Hospital Virgen de los Lirios de Alcoy (Alicante)
12. Internal Medicine Department. Denia Hospital (Alicante).
13. Microbiology Department. Hospital Vega Baja (Orihuela, Alicante)-FISABIO.
14. Microbiology Department. University Hospital San Juan de Alicante (San Juan, Alicante)-FISABIO.
15. Internal Medicine Department. Torrevieja University Hospital (Torrevieja, Alicante).
16. Microbiology Department. Elda General University Hospital (Elda, Alicante)-FISABIO.
17. Microbiology Department. Hospital Virgen de los Lirios de Alcoy (Alicante)
18. Microbiology Department. Denia Hospital (Alicante).
19. Microbiology Department. Alicante General University Hospital (Alicante, Spain). ISABIAL
20. **Microbiology Department. Elche General University Hospital (Elche, Spain). FISABIO**
21. Department of Public Health, Science History and Gynaecology. University Miguel Hernández de Elche, Alicante, Spain

Thank you again for your time and help to improve our paper.

Yours sincerely,

Jara Llenas-García, on behalf of all authors.

**CONTACT ADDRESSES**

| Corresponding author:  Jara Llenas-García, PhD  Hospital Vega Baja-FISABIO Crta Orihuela-Almoradí s/n  03340 Orihuela (Alicante) Spain  Telephone number: +34652584257  Email: jarallenas@gmail.com |  |
| --- | --- |
