# Supplementary material for: Chagas disease screening in pregnant Latin American women: Adherence to a systematic screening protocol in a non-endemic country
Source: PLoS Negl Trop Dis. 2021 Mar 24;15(3):e0009281. doi: 10.1371/journal.pntd.0009281 (PMC8021187; doi:10.1371/journal.pntd.0009281)
Supplement: Attachment — Submitted filename: 2 Letter to editors with answers to reviewers.docx [file pntd.0009281.s004.docx]

Jara Llenas-García, PhD

Hospital Vega Baja-FISABIO
Crta Orihuela-Almoradí s/n
03340 Orihuela (Alicante) Spain

Telephone number: +34652584257

Email: jarallenas@gmail.com

Erica Silberstein

Guest Editor

PLOS Neglected Tropical Diseases

Alain Debrabant

Deputy Editor

PLOS Neglected Tropical Diseases

17th February 2021

**To the editors of PLOS Neglected Tropical Diseases**

**Second response-to-reviewers letter and revised manuscript entitled “Chagas disease screening in pregnant Latin American women: systematic screening protocol in a non-endemic country" Ref: PNTD-D-20-01929R1**

Dear Editors,

This document provides point-by-point responses to the reviewers’ comments. Their comments, reproduced in *italics*, are followed by our responses and, finally by the additional text and changes (in red) to the manuscript first submitted. We thank again the three reviewers for their comments which have much helped improve our paper.

*Reviewer #1: Globally, we endorse the revised version of the MS PNTD-D-20-01929R1. However, we can hardly accept that the authors do not add a reference directly related to the MS topic, giving as pretext they could not read the proposed document, as mentioned:*

*“Reference Chap 23, p517-559 in American trypanosomiasis-Chagas disease. One hundred years of research. Elsevier, 2017 ,2 edition, ISBN 978-0-12-801029-7 has not been added because unfortunately we could not read the proposed chapter”.*

*It is the duty of authors to gather all references to build their paper (it is always possible to ask the paper directly to its authors). So, publication of this MS R1 can be accepted if such reference is added (e.g. l. 109, 110, 323) (the corresponding reference paper is sent by separated email).*

- We thank the reviewer for the reference chapter. Reference has been added in line 110:

“However, prompt treatment with anti-parasitic therapy can achieve cure in most cases[15,16].”

- And in line 323:

“There is also a need to standardize, expand and reinforce CD screening in all women of Latin American origin who are of childbearing age (pregnant or not); treating childbearing age women is a useful strategy to decrease vertical transmission in potential future pregnancies, since there is a correlation between the parasitemia of the mother and congenital infection[16,48,49].”

*Reviewer #3: Trypanosoma cruzi should be in italic in the references (ref.6,12,13,14,17,20,22,30,35,47,48)*

- *Trypanosoma cruzi* has been written in italics in all references mentioned:
  - *Ref. 6* , line 379. “Treatment of Women Infected with *Trypanosoma cruzi* and Its Effect on Preventing”
  - *Ref.12, line 398. “*disease in pregnant women and congenital transmission of *Trypanosoma cruzi* in the”
  - *Ref. 13, line 402. “Trypanosoma cruzi* infection in Latin American pregnant women living outside endemic”
  - *Ref.14, line 406.”…* transmission of *Trypanosoma cruzi*: A systematic review and meta-analysis. BJOG: An..”
  - *Ref.18 (former17), line 420. “*Screening for *Trypanosoma cruzi* infection in immigrants and refugees: Systematic..”
  - *Ref. 21 (former 20), line 432. “*Brusola A, et al. Prevalence of *Trypanosoma cruzi* infection in pregnant Latin American..”
  - *Ref. 23 (former 22), line 440. “*Prevalence of *Trypanosoma cruzi* infection in Latin American pregnant women and level”
  - *Ref. 31 (former 30), line 467. “*Guruceta MI, Egurbide Arberas MV. Prevalence of *Trypanosoma cruzi* infection and..”
  - *Ref.36 (former 35), line 486. “*transmission of *Trypanosoma cruzi* in non-endemic areas: Evaluation of a screening”
  - *Ref.44 (former 43), line 515. “*of *Trypanosoma cruzi*: An operational outline for detecting and treating infected infants”
  - *Ref. 48 (former 47), line 529. “*Detectable *Trypanosoma cruzi* parasitemia during pregnancy and delivery as a risk”
  - *Ref. 49 (former 48), line 532. “*Carlier Y, Schijman AG, Kemmerling U. Placenta, *Trypanosoma cruzi*, and Congenital..”

*Ref 8 delete Rodrigues MM, editor*

- Rodrigues MM, editor has been deleted (line 387)

“Requena-Méndez A, Aldasoro E, de Lazzari E, Sicuri E, Brown M, Moore DAJ, et al. Prevalence of Chagas disease in Latin-American migrants living in Europe: a systematic review and meta-analysis. PLoS Negl Trop Dis. 2015;9: e0003540. doi:10.1371/journal.pntd.0003540”

*Ref 10 name of the Journal should be abbreviated, delete public library of science*

- Name of the journal has been abbreviated and public library of science has been deleted. Volume, number and e page has been added (lines 394).

“Carlier Y, Altcheh J, Angheben A, Freilij H, Luquetti AO, Schijman AG, et al. Congenital Chagas disease: Updated recommendations for prevention, diagnosis, treatment, and follow-up of newborns and siblings, girls, women of childbearing age, and pregnant women. PLoS Negl Trop Dis. 2019; 13(10):e0007694. doi:10.1371/journal.pntd.0007694”

*Ref 13 year*

- Year has been added (line 404)

“Colombo V, Giacomelli A, Casazza G, Galimberti L, Bonazzetti C, Sabaini F, et al. Trypanosoma cruzi infection in Latin American pregnant women living outside endemic countries and frequency of congenital transmission: a systematic review and meta-analysis. J Travel Med. 2021; 28(1): taaa170. doi:10.1093/jtm/taaa170”

*Ref 14 volume*

- Volume has been added (line 407)

“Howard EJ, Xiong X, Carlier Y, Sosa-Estani S, Buekens P. Frequency of the congenital transmission of *Trypanosoma cruzi*: A systematic review and meta-analysis. BJOG: An International Journal of Obstetrics and Gynaecology. 2014; 121(1): 22–33. doi:10.1111/1471-0528.12396”

*Ref 42 Chagas (Capital letter for the initial)*

- Now ref. 43 (former 42). Chagas has been written with Capital letter for the initial (line 511-12)

“Ramos-Rincón JM, Mira-Solves JJ, Ramos-Sesma V, Torrús-Tendero D, Llenas-García J, Navarro M. Healthcare professionals and students’ awareness of Chagas disease: Design and validation of Chagas level of knowledge scale (ChaLKS). Am J Trop Med Hyg. 2020;103: 437–44. doi:10.4269/ajtmh.19-0677”

Thank you once more for your time and help to improve our paper.

Yours sincerely,

Jara Llenas-García, on behalf of all authors.

**CONTACT ADDRESSES**

| Corresponding author:  Jara Llenas-García, PhD  Hospital Vega Baja-FISABIO Crta Orihuela-Almoradí s/n  03340 Orihuela (Alicante) Spain  Telephone number: +34652584257  Email: jarallenas@gmail.com |  |
| --- | --- |
